# An HDAC2-TET1 switch at distinct chromatin regions significantly promotes the maturation of pre-iPS to iPS cells

**DOI:** 10.1093/nar/gkv430

**Published:** 2015-05-01

**Authors:** Tingyi Wei, Wen Chen, Xiukun Wang, Man Zhang, Jiayu Chen, Songcheng Zhu, Long Chen, Dandan Yang, Guiying Wang, Wenwen Jia, Yangyang Yu, Tao Duan, Minjuan Wu, Houqi Liu, Shaorong Gao, Jiuhong Kang

**Affiliations:** 1 Clinical and Translational Research Center of Shanghai First Maternity & Infant Hospital, Shanghai Key Laboratory of Signaling and Disease Research, School of Life Science and Technology, Tongji University, 1239 Siping Road, Shanghai 200092, PR China; 2 Group of Epigenetic Reprogramming, State Key Laboratory of Cell Biology, Institute of Biochemistry and Cell Biology, Shanghai Institutes for Biological Sciences, Chinese Academy of Sciences, Shanghai 200031, China; 3 Department of Embryology and Histology, Second Military Medical University, Shanghai 200433, PR China

## Abstract

The maturation of induced pluripotent stem cells (iPS) is one of the limiting steps of somatic cell reprogramming, but the underlying mechanism is largely unknown. Here, we reported that knockdown of histone deacetylase 2 (HDAC2) specifically promoted the maturation of iPS cells. Further studies showed that HDAC2 knockdown significantly increased histone acetylation, facilitated TET1 binding and DNA demethylation at the promoters of iPS cell maturation-related genes during the transition of pre-iPS cells to a fully reprogrammed state. We also found that HDAC2 competed with TET1 in the binding of the RbAp46 protein at the promoters of maturation genes and knockdown of TET1 markedly prevented the activation of these genes. Collectively, our data not only demonstrated a novel intrinsic mechanism that the HDAC2-TET1 switch critically regulates iPS cell maturation, but also revealed an underlying mechanism of the interplay between histone acetylation and DNA demethylation in gene regulation.

## INTRODUCTION

Induced pluripotent stem (iPS) cells have been generated from a number of cell types via the enforced expression of the ‘OSKM’ group of transcription factors: Oct4, Sox2, Klf4 and c-Myc (1,2). It has been shown that OSKM-induced somatic cell reprogramming is a multi-step process involving initiation, maturation and stabilization (3). One important event in the initiation phase of reprogramming is an early strong induction of the mesenchymal-to-epithelial transition (MET), which is characterized by the upregulation of epithelial components and morphological transformation into epithelial-like colonies (4), followed by the appearance of AP- and SSEA1-positive cells in the cultured colonies (5). Studies have shown that both bone morphogenetic protein (BMP) agonists and transforming growth factor β (TGF-β) inhibitors increase reprogramming efficiency by favoring the MET (3,6). Our previous studies also found that the miR-29b and the miR-200 families significantly promoted the initiation event of reprogramming by upregulating the expression of MET-related genes (7,8). To date, a considerable number of reprogramming studies have examined the transcription factors, signaling pathways and miRNAs that regulate the initiation of iPS cell generation; however, relatively little is known about the maturation of iPS cell. Recent data have demonstrated that the maturation of iPS cells, which is characterized by high expression levels of genes such as *Nanog*, *Sall4*, *Esrrb*, *Cripto*, *Rex1* and *Tcl1* (9–13), is the limiting step in the direct reprogramming of human fibroblasts toward pluripotency (14). Thus, identifying the mechanisms underlying the maturation of iPS cells is critically important.

Unlike Oct4, Nanog is dispensable for the combinations of exogenous factors that have been found to convert mouse somatic cells into iPS cells (1). Somatic cells that cannot produce Nanog still undergo the early stage of the reprogramming process; however, in *Nanog*^−/−^ pre-iPS cells, the transition to the pluripotent state is prevented. This situation is similar to that of the E3.5 inner cell mass (ICM), where Oct4 and Sox2, but not Nanog, are ubiquitously expressed prior to the appearance of the naïve epiblast (15). Pre-iPS cells exhibit embryonic stem (ES)-like morphology and the downregulation of somatic cell marker gene expression, but they are characterized by the incomplete silencing of retroviral transgenes, non-responsiveness to LIF and, most crucially, an inability to form chimeras. Nanog drives the broad changes in the transcriptional program that are associated with the acquisition of pluripotency and these transcriptional changes can promote the transition from pre-iPS cells to pluripotent iPS cells. Several studies have indicated that Nanog expression in pre-iPS cells is weak or absent and can be activated by treatment with 2i (PD0325901, an inhibitor of MAP kinase; CHIR99021, an inhibitor of glycogen synthase kinase 3 β)/LIF (16,17). The downregulation of endogenous Gata4 by short hairpin RNAs during reprogramming has been shown to both augment the mRNA levels of endogenous *Nanog* and increase the efficiency of the reprogramming process (12). These studies indicate the importance of Nanog as a key factor in the maturation of iPS cells; however, the mechanisms underlying the activation of *Nanog* and other maturation phase-related genes during iPS cell generation remain largely unclear.

The efficiency of the reprogramming induced by the four OSKM factors can be improved significantly by treatment with small-molecule inhibitors of intrinsic histone deacetylases (HDACs), of which valproic acid (VPA), a specific inhibitor of class I and II HDACs, is the most potent to be reported to date (18). Furthermore, a combination of VPA and three other small chemicals is sufficient to induce reprogramming by a single transcription factor, Oct4 (19). The most recent study also reported that low levels of *Hdac2* or the suppression of *Hdac2* expression was required for highly efficient somatic reprogramming by the miR302/367 cluster (20). These discoveries suggest that HDACs might function as critical epigenetic barriers to reprogramming by repressing the establishment of a transcriptional network that controls pluripotency. However, the specific roles of distinct HDACs and the factors that act downstream of HDAC inhibition in the activation of maturation phase-related genes and iPS cell maturation remain unknown.

An emerging role for DNA demethylation in the generation of iPS cells has been reported. DNA methyltransferase inhibitors significantly improve reprogramming efficiency (18). The formation of 5-hydroxymethylcytosine (5hmc) via the hydroxylation of 5-methylcytosine (5mc) by the Tet (ten-eleven translocation) family of methylcytosine hydroxylases, which includes three members (*Tet1*, *Tet2* and *Tet3*), has been detected in many cell types and is involved in active DNA demethylation (21–25). It has been proposed that active DNA demethylation plays an important role in reactivating pluripotency-related genes, which are hypermethylated and silenced in somatic cells (26). A recent study showed that the DNA hydroxylase Tet1 promoted reprogramming to pluripotency and could, in fact, replace exogenous Oct4 in this process (27). The promoter regions of maturation phase-related genes are highly methylated in somatic cells and their activation during iPS cell generation is discovered in a pioneering iPS cell study in 2006 (1). Both histone hyperacetylation and DNA demethylation are generally required for the activation and transcription of silent genes, especially in cases of silencing mediated by DNA methylation (28). Therefore, determining whether and how HDACs and TET family proteins function in activating iPS cell maturation genes is critical for fully understanding somatic cell reprogramming.

In the present study, we showed that knockdown of *Hdac2* specifically promoted the maturation of iPS cells. Furthermore, we characterized the HDAC2-TET1 switch at distinct chromatin regions as a novel intrinsic modulator of iPS cell maturation and one mechanism of the interplay between histone acetylation and DNA demethylation.

## MATERIALS AND METHODS

### Cell culture and iPS cell induction

OG-MEFs were derived from transgenic mice at E13.5 and were maintained in Dulbecco's modified Eagle's medium (DMEM, Gibco) supplemented with high glucose, 1×nonessential amino acids (NEAA, Thermo), 1×L-glutamine (Thermo), β-mercaptoethanol (Gibco) and 10% fetal bovine serum (FBS). All the MEFs used for these experiments were pooled and collected before passage 3. The methods of maintaining plat-E cells and feeder cells and the viral infection methods and iPS cell induction were as previously described (1). iPS cells and mouse ES cells were maintained in knockout-DMEM medium (Gibco, N.Y, USA) containing 20% knockout serum replacement (KOSR) (Gibco, N.Y, USA), 1×Penicillin/Streptomycin Solution (P/S) (Hyclone), 1×NEAA (Thermo), 1×L-glutamine (Thermo) and β-mercaptoethanol (Gibco) with leukemia-inhibitory factor (LIF, 10 000×, Millipore). iPS cells were maintained on feeder layers of mitomycin C (Sigma)-treated MEFs.

### Transfection

Transfection was performed using P3 Primary Cell 4D-Nucleofector™ X Kit (Lonza) according to the manufacturer's protocol. Briefly, 2.5×10^5^ cells were resuspended in 20 μl of P3 4D-Nucleofector™ X Solution contained 0.6 μg plasmid. After nucleofection, cells were maintained on feeder layers.

### *In* *vitro* differentiation of iPS cells and teratoma formation

For embryoid body (EB) formation, iPS cells were trypsinized into a single-cell suspension and the hanging drop method was used to generate EBs. Each drop contained 1×10^3^ iPS cells in 20 μl of KOSR medium without LIF. EBs were cultured in hanging drops for 3 days before being reseeded into gelatin-coated 48-well plates and incubated for another 6 days. RNA was extracted from the EBs for the analysis of differentiation markers. To generate teratomas, iPS cells were trypsinized, resuspended at a concentration of 2×10^6^ cells/150 μl and injected into nonobese diabetic/severe combined immunodeficient (NOD-SCID) mice. The mice were examined for tumor formation every week for up to 3–4 weeks. Tumors were harvested and fixed in a formaldehyde solution for 24 h at room temperature before paraffin embedding and subsequent HE staining.

### Chimera generation

The specific pathogen-free mice were housed in the animal facility of the Tongji University. All of our study procedures were consistent with the Tongji University Guide for the care and use of laboratory animals. To produce chimeric mice, 10–15 iPS cells, pre-iPS cells or coverted iPS cells were subsequently injected into the cavity of the Institute of Cancer Research (ICR) blastocysts and transplanted into the oviducts of pseudo-pregnant mice 2–3 h later.

### Chromatin immunoprecipitation (ChIP)

The Chromatin immunoprecipitation (ChIP) assay was performed using the EZ ChIP kit (Millipore) according to the manufacturer's protocol. Briefly, 5×10^7^ cells were used for each immunoprecipitation. The cells were fixed with 1% formaldehyde, collected, resuspended in ChIP lysis buffer (1% Sodium dodecyl sulphate (SDS), 10 mM Ethylenediaminetetraacetic acid (EDTA) and 50 mM Tris–HCl, pH 8.1) and sonicated using the Qsonica Sonifier to generate fragments of 750 to 1000 base pairs (bp). Soluble chromatin was diluted threefold in ChIP dilution buffer (10 mM Tris–HCl at pH 7.5, 140 mM NaCl, 1 mM EDTA, 0.5 mM EGTA, 1% Triton X-100, 0.1% SDS and 0.1% sodium deoxycholate) and incubated with Protein G agarose (Millipore) coupled to the specific antibody. After incubation, the immunocomplexes were washed sequentially with a low-salt wash buffer (0.1% SDS, 1% Triton X-100, 2 mM EDTA, 20 mM Tris–HCl at pH 8.1 and 150 mM NaCl), a high-salt wash buffer (0.1% SDS, 1% Triton X-100, 2 mM EDTA, 20 mM Tris–HCl at pH 8.1 and 500 mM NaCl), an LiCl wash buffer (0.25 M LiCl, 1% NP-40, 1% deoxycholate, 1 mM EDTA and 10 mM Tris–HCl at pH 8.1) and TE buffer (10 mM Tris–l at pH 7.5 and 1 mM EDTA). Immunocomplexes were eluted in ChIP elution buffer (1% SDS and 0.1 M NaHCO_3_) and the crosslinking was reversed overnight at 65°C. Samples were treated with Proteinase K and RNase A, extracted with phenol/chloroform and precipitated with ethanol. Purified chromatin was quantified using qPCR with SYBR Green on an Mx3000P with the primers in Supplementary Table S1. The fold enrichment of the immunoprecipitated samples normalized for input was determined relative to control IgG (Millipore). The antibodies used in the ChIP assay are listed in Supplementary Table S2.

### Tetraploid complementation assay (TCA)

Tetraploid complementation assay (TCA) was performed as followed. Briefly, diploid blastocysts were collected from the uterus of E3.5 superovulated ICR females. To generate mice by tetraploid embryo complementation, two-cell embryos were collected from the oviducts of ICR females and electrofused to produce one-cell tetraploid embryos. Approximately 15 iPS cells were injected into the tetraploid blastocyst cavity. The injected blastocysts were transferred to each uterine horn of 2.5-days-postcoitum pseudopregnant ICR females. Pregnant recipients with tetraploid embryos were subjected to caesarean section on day 18.5 of gestation.

## RESULTS

### Knockdown of *Hdac2* improves OSKM-mediated iPS cell generation

**Figure 1.**
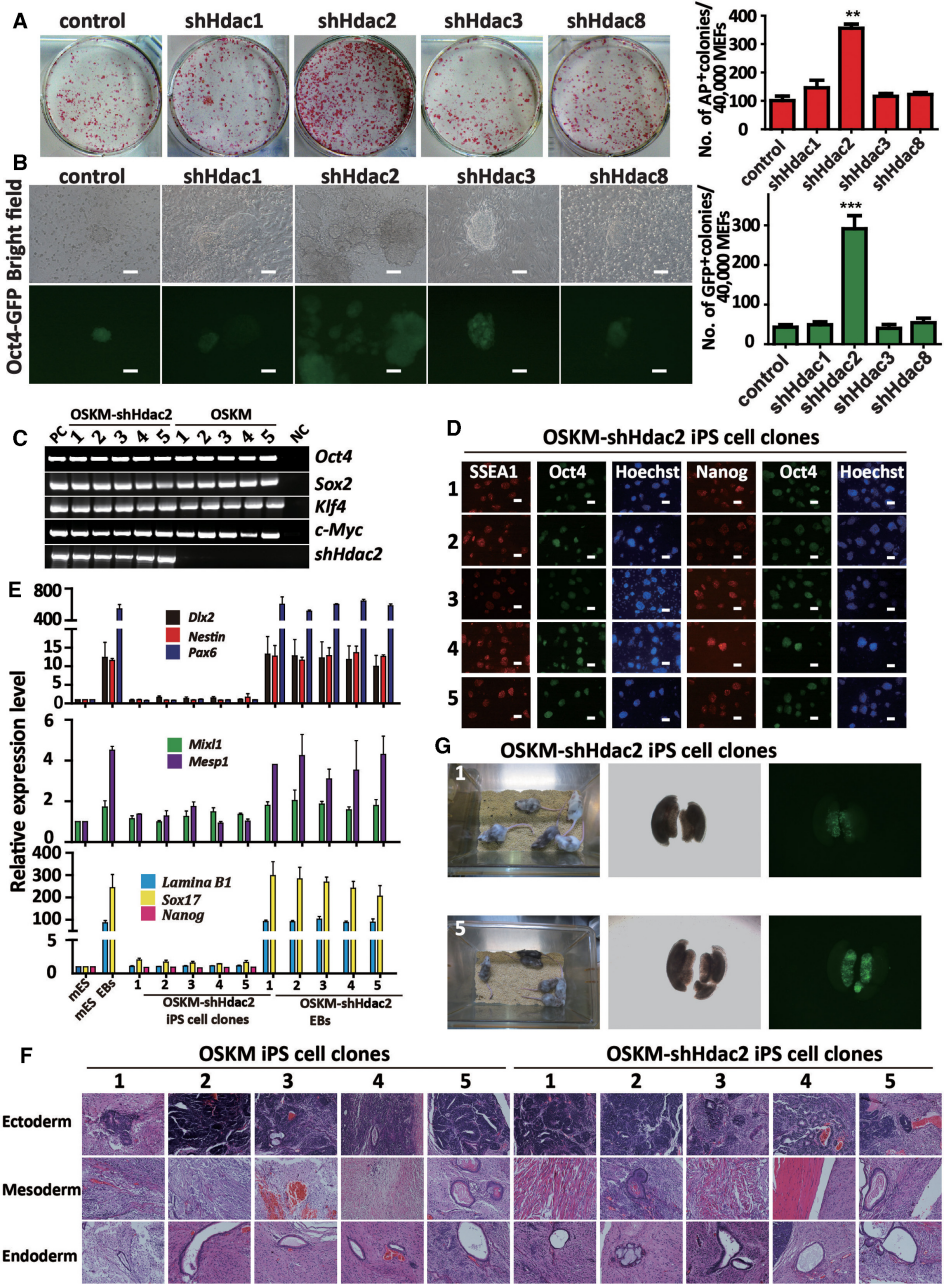
Knockdown of *Hdac2* improves the generation of OSKM-iPS cells. (**A**) AP staining (on reprogramming day 8) of colonies from OG-MEFs transduced with OSKM in combination with control shRNA, *shHdac1*, *shHdac2*, *shHdac3* or *shHdac8*. AP-positive colony numbers are shown on the right of the figure. (**B**) The morphology of typical Oct4-GFP iPS cell colonies on reprogramming day 12 in combination with *shHdac1*, *shHdac2*, *shHdac3* or *shHdac8*, respectively. GFP-positive colony numbers are shown on the right. Scale bar, 100 μm. (**C**) Genomic PCR analyses showed that the OSKM-shHdac2-derived iPS cell clones (1–5) were infected with OSKM and *shHdac2* simultaneously. OG-MEFs were used as a negative control (NC) and OG-MEFs infected with the OSKM viruses or *shHdac2* were used as positive controls (PC). (**D**) Immunostaining for pluripotency markers (Oct4, Nanog and SSEA-1) in the OSKM-shHdac2-derived iPS cell clones. Scale bar, 100 μm. (**E**) qRT-PCR analyses of markers for all three germ layers (*Dlx2, Nestin* and *Pax6* for ectoderm, *Mixl1* and *Mesp1* for mesoderm, and *Lamina B1* and *Sox17* for endoderm) and a pluripotency factor (*Nanog*) in mES cells, mES EBs, OSKM-shHdac2 iPS cell clones and OSKM-shHdac2 EBs. The expression levels were normalized to *Gapdh*. (**F**) OSKM-shHdac2-derived iPS cell clones induced teratomas containing all three embryonic germ layers. Representative images of HE staining for neural tissue (ectoderm), skeletal muscle (mesoderm) and epithelial tissue (endoderm) are shown. (**G**) Two-week-old chimeric mice derived from OSKM-shHdac2-1and OSKM-shHdac2-5 iPS cell clones (C57BL/6 background). Oct4-GFP positive cells derived from OSKM-shHdac2-1 or OSKM-shHdac2-5 iPS cell clone are present in the genital ridge of a female embryo at 13.5 day postcoitum (dpc). The data in a, b and e represent the means ± S. E. M. of three independent experiments. **P < 0.01, ***P < 0.001 (two-tailed Student's *t*-test).

Previous studies have shown that the inhibition of class I HDACs significantly improved the efficiency of iPS cell generation (18). Hence, we constructed retroviral vectors expressing shRNAs that specifically targeted mouse *Hdac1*, *Hdac2*, *Hdac3* and *Hdac8*. The efficiency of knockdown by the individual viruses in MEFs was confirmed by using both quantitative reverse transcriptase PCR (qRT-PCR, Supplementary Figure S1a) and western blot (Supplementary Figure S1b). MEFs containing *Oct4* promoter-driven green fluorescent protein (OG-MEFs) were obtained from an Oct4-GFP transgenic mouse and iPS cells were generated from these cells (18,29). We infected OG-MEFs with the OSKM factors in combination with the shRNA viruses corresponding to *Hdac1*, *Hdac2*, *Hdac3* and *Hdac8*. On day 8 post-infection, we found that cells infected with viruses containing OSKM and *Hdac2* shRNA (*shHdac2*) had formed approximately 4-fold as many AP-positive colonies as the cells infected with viruses containing OSKM and a control shRNA (Figure 1a). We further quantified the number of Oct4-GFP-positive colonies with mES cell-like morphology, which has been shown to be a stringent method of identifying reprogramming (30). Compared with the OSKM group, the knockdown of *Hdac2* increased the number of Oct4-GFP-positive colonies by approximately 7-fold (Figure 1b), whereas the knockdown of *Hdac1*, *Hdac3* or *Hdac8* had no significant effect on the numbers of AP-positive and Oct4-GFP-positive colonies. Furthermore, our results showed that the effect of *Hda*c*2* knockdown alone is slightly lower than that of the class I HDACs combinatorial knockdown on reprogramming (Supplementary Figure S2a-2c). *Hdac2* knockdown accelerated reprogramming (Supplementary Figure S3a and S3b), whereas *Hdac2* overexpression significantly inhibited reprogramming (Supplementary Figure S4a and S4b). Overall, these data indicated that HDAC2 is the major HDAC involved in the repression of reprogramming.

In addition, the morphology of the iPS cells induced by OSKM plus *shHdac2* viruses (OSKM-shHdac2-iPS cells) was similar to that of mES clone. The integration of exogenous OSKM factors and the shRNA targeting *Hdac2* was examined by using PCR (Figure 1c). The introduced exogenous genes had been silenced (Supplementary Figure S1c) at the second passage. qRT-PCR using five different OSKM-shHdac2-iPS cell clones revealed that they all expressed pluripotent markers (endogenous *Oct4: endo*-*Oct4*, endogenous *Sox2: endo*-*Sox2*, *Nanog* and *Essrb*) at levels equivalent to OSKM-iPS cell clones and mES cells (Supplementary Figure S1d). Simultaneously, we examined and found the endogenous expression of Nanog and SSEA1 in the OSKM-shHdac2-iPS cell clones at the second passage (Figure 1d). To investigate whether the derived clones exhibited the full differentiation potential, we used floating cultivation to form EBs. After 3 days in suspension culture, the shHdac2-derived clones developed into spherical structures, indicating efficient EB formation. Then, these EBs were transferred to gelatin-coated plates and cultivated for 6 days. qRT-PCR confirmed that these differentiated EBs expressed relevant markers for all three germ layers. In contrast, the expression of pluripotency markers such as *Nanog* was markedly decreased (Figure 1e).

In addition, OSKM-shHdac2-derived iPS cell clones were injected into the dorsal flanks of athymic nude mice (NOD-SCID) to assess their ability to form teratomas. OSKM-iPS cell clones were injected as control. Teratomas were readily observed 4 weeks after cells injection and a subsequent histological analysis showed that the tumors generated by the OSKM-shHdac2-derived iPS cell clones contained neural tissue (ectoderm), skeletal muscle (mesoderm), blood (mesoderm) and epithelial tissue (endoderm) (Figure 1f). Further, to test whether OSKM-shHdac2-derived iPS cell clones could contribute to the germline of mice, we injected two different OSKM-shHdac2-derived iPS cell clones derived from Oct4-GFP MEFs into blastocyst. Live chimeras with high coat color contribution were obtained (Figure 1g). Mouse gonads were collected at E14.5 and visualized by whole mount fluorescence for GFP expression. Both clones contributed efficiently to germ cells in the gonads of chimeric mice (Figure 1g). Taken together, our results showed that endogenous HDAC2 plays a negative role in OSKM-mediated mouse somatic cell reprogramming and the knockdown of *Hdac2* improved the efficiency of OSKM-induced reprogramming without sacrificing the pluripotency and differentiation capability of iPS cells.

### Knockdown of *Hdac2* converts pre-iPS cells into fully reprogrammed iPS cells

**Figure 2.**
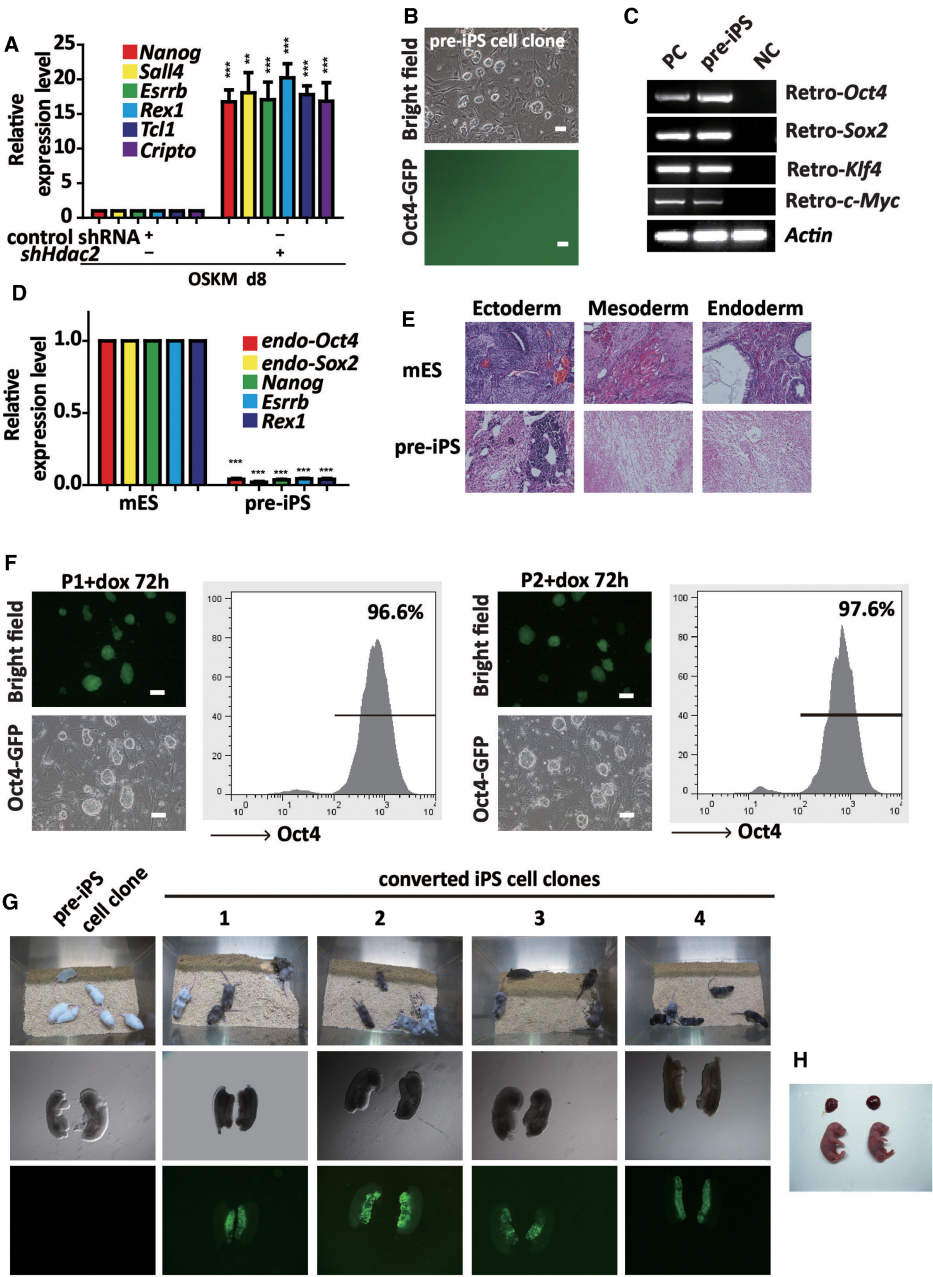
Knockdown of *Hdac2* promotes the further reprogramming of pre-iPS cells. (**A**) qRT-PCR analyses of maturation phase-related genes in MEFs under reprogramming (day 8) infected with OSKM in combination with control shRNA or *shHdac2*. (**B**) Phase contrast (upper panel) and fluorescence (lower panel) microscopic images showing the morphology of a typical mES-like but GFP-negative pre-iPS cell clone. Scale bar, 100 μm. (**C**) RT-PCR was performed to confirm *Oct4*, *Sox2*, *Klf4* and *c-Myc* transgene expression in the pre-iPS cells. OG-MEFs were used as a negative control (NC) and OG-MEFs infected with the OSKM viruses were used as positive controls (PC). (**D**) qRT-PCR analyses of the pluripotency genes in the pre-iPS cells. The mRNA levels are shown relative to the mES cells. *Actin* was used as an internal control. (**E**) HE staining of teratomas derived from mES cells and pre-iPS cells. (**F**) Morphology of P1 and P2 clones by dox-inducible *Hdac2* knockdown after treatment with dox for 72 h. A FACS analysis showed the proportion of Oct4-GFP cells after dox treatment. Scale bar, 100 μm. (**G**) Two-week-old chimeric mice derived from P1 and four converted iPS cell clones (1, 2, 3 and 4; C57BL/6 background). (**H**) Two live-born (E18.5) all-iPS cell mice generated from C4-iPS cells via tetraploid complementation assay (TCA). The data in a and d represent the means ± S. E. M. of three independent experiments. **P < 0.01, ***P < 0.001 (two-tailed Student's *t*-test).

The results showed that the expression of the maturation phase-related genes (*Nanog*, *Sall4*, *Esrrb*, *Rex1*, *Tcl1* and *Cripto*) was significantly increased in the cells that were induced with a combination of OSKM and *shHdac2*, compared with those that were induced using OSKM and control shRNA (Figure 2a). The expression of reprogramming initiation phase genes (*Cdh1*, *Ocln*, *Epcam*, *Zeb1* and *Snail*) was not upregulated (Supplementary Figure S5a). Single pre-iPS cell clone was picked up and expanded. We found that these pre-iPS cells formed colonies with mES-like morphology and had integrated exogenous OSKM factors (Supplementary Figure S5b), but they were Oct4-GFP negative (Figure 2b). Oct4-GFP was not activated in these pre-iPS cells, even if the cells were split for over 30 passages on the feeder cells. The introduced exogenous genes had not been silenced (Figure 2c). Compared with the mES cells, the pre-iPS cells expressed lower levels of pluripotency markers (Figure 2d). Moreover, the teratomas grown from the pre-iPS cells contained immature ectoderm with a rosette-like neural tube, mesoderm with a loose texture and abnormal endoderm with epithelial dysplasia (Figure 2e). We infected pre-iPS cells with dox-inducible *shHdac2–1* or *shHdac2–2* lentvirus, and then a single clone containing dox-inducible *shHdac2–1* or *shHdac2–2* was selected. After treatment with dox for 72 h, the pre-iPS cells containing dox-inducible *shHdac2–1* or *shHdac2–2* (hereafter called P1 and P2, respectively) had become Oct4-GFP-positive (Figure 2f), indicating that the activation of the *Oct4* promoter and the full reprogramming of iPS cells had been achieved. The Oct4-GFP-positive iPS clones converted from P1 and P2 by downregulation of *Hdac2* (hereafter called converted iPS cells; C1, C2, C3 and C4) were picked up. The gender of the four conditional iPS cell lines (1, 2, 4 and 5) and three OSKM-shHdac2 iPS cell lines (2, 3 and 4) was male, while the gender of the others was female (Supplementary Figure S5c). To identify the contribution of the pre-iPS cells and the converted iPS cells to germline cells of mice, we injected them into blastocyst. The pre-iPS cell clone did not yield functional germline cells of chimeras. In contrast, live chimeras with high coat color contribution from four different converted iPS cell clones were obtained (Figure 2g). Mouse gonads were collected at E14.5 and visualized by whole mount fluorescence for GFP expression. All four clones contributed efficiently to germ cells in the gonads of chimeric mice (Figure 2g). Further, we tested the *in vivo* developmental potential of the converted iPS cells by TCA, which is the most stringent assay for pluripotency. The converted iPS cells gave rise to viable all-iPS cell derived pups (Figure 2h). Collectively, the results showed that the knockdown of *Hdac2* could efficiently convert pre-iPS cells into mature iPS cells and at the molecular level, *Hdac2* expression was negatively correlated with the expression of maturation phase-related genes.

### HDAC2 impedes the transcriptional activation of maturation phase-related genes and the maturation of iPS cells

**Figure 3.**
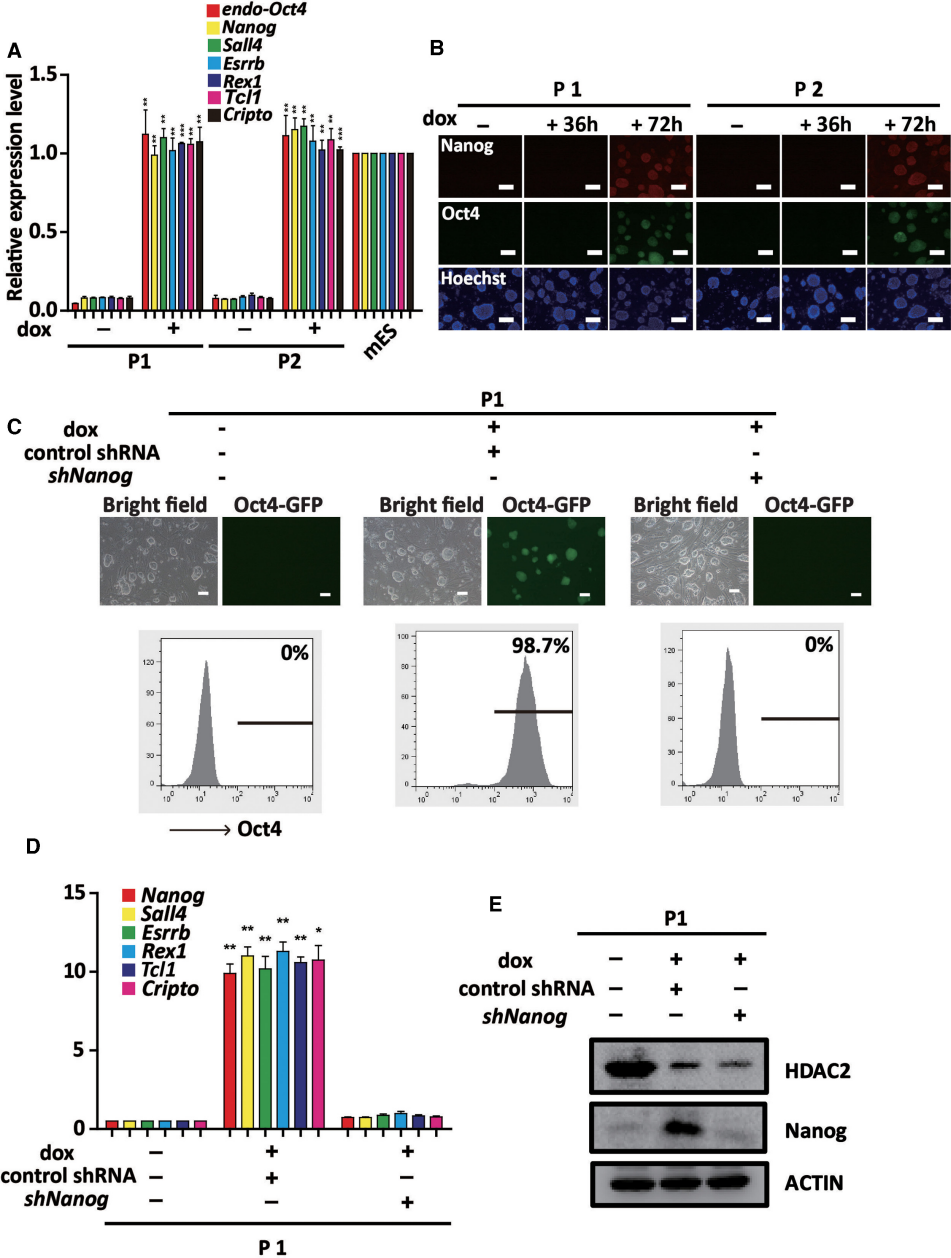
HDAC2 blocks reprogramming at the pre-iPS cell stage by impeding the transcriptional activation of maturation phase-related genes. (**A**) qRT-PCR analyses of *Oct4* and the maturation phase-related genes in P1 and P2 clones treated without or with dox for 72 h. (**B**) Immunostaining for Nanog and Oct4-GFP reporter activity in P1 and P2 clones treated with dox for 36 and 72 h. (**C**) Phase contrast and ﬂuorescence images of P1 clone transfected with control shRNA and *shNanog* after dox treatment. Scale bar, 100 μm. Flow cytometry analysis of Oct4-GFP reporter activity (below). (**D**) qRT-PCR analysis of maturation phase genes (*Nanog*, *Sall4*, *Esrrb*, *Rex1*, *Tcl1* and *Cripto*) in P1 clone transfected with control shRNA and *shNanog* after dox treatment. *Gapdh* was used as an internal control. (**E**) Representative western blot for HDAC2 and Nanog expression in P1 clone transfected with control shRNA or *shNanog* upon dox treatment. The data in a and d represent the means ± S. E. M. of three independent experiments. *P < 0.05, **P < 0.01, ***P < 0.001 (two-tailed Student's *t*-test).

We found that the expression of the maturation phase-related genes (*Nanog, Sall4, Esrrb, Rex1, Tcl1* and *Cripto*) was significantly increased in P1 and P2 clones after 72 h dox treatment (Figure 3a). The expression of Nanog was also confirmed at different time by immunostaining in two pre-iPS cell clones (Figure 3b). We further addressed the role of *Nanog* activation in the *Hdac2* knockdown-induced transition of pre-iPS cells to iPS cells, and showed that knockdown of the *Nanog* in pre-iPS cells impaired the pre-iPS to iPS cell transition (Figure 3c). Moreover, the expression of the maturation genes was not activated (Figure 3d). The knockdown efficiency of HDAC2 and Nanog was confirmed by western blot analyses (Figure 3e). A previous study has shown that treating pre-iPS cells with 2i resulted in the activation of endogenous *Nanog* (17). Consistently, after 2i/LIF treatment for 7 days, stable Oct4-GFP expression appeared in the P1 cells (Supplementary Figure S6a), accompanied by the decreased expression of HDAC2 and the upregulated expression of maturation phase-related genes (Supplementary Figure S6b and S6c). Interestingly, the 2i-induced transition could be significantly blocked by the knockdown of *Hdac2* (Supplementary Figure S6d). Collectively, these results suggested that the activation of Nanog played a critical role in the maturation of pre-iPS cells mediated by *shHdac2*.

### Histone acetylation is primarily required for DNA demethylation and maturation phase-related gene activation during iPS cell maturation

**Figure 4.**
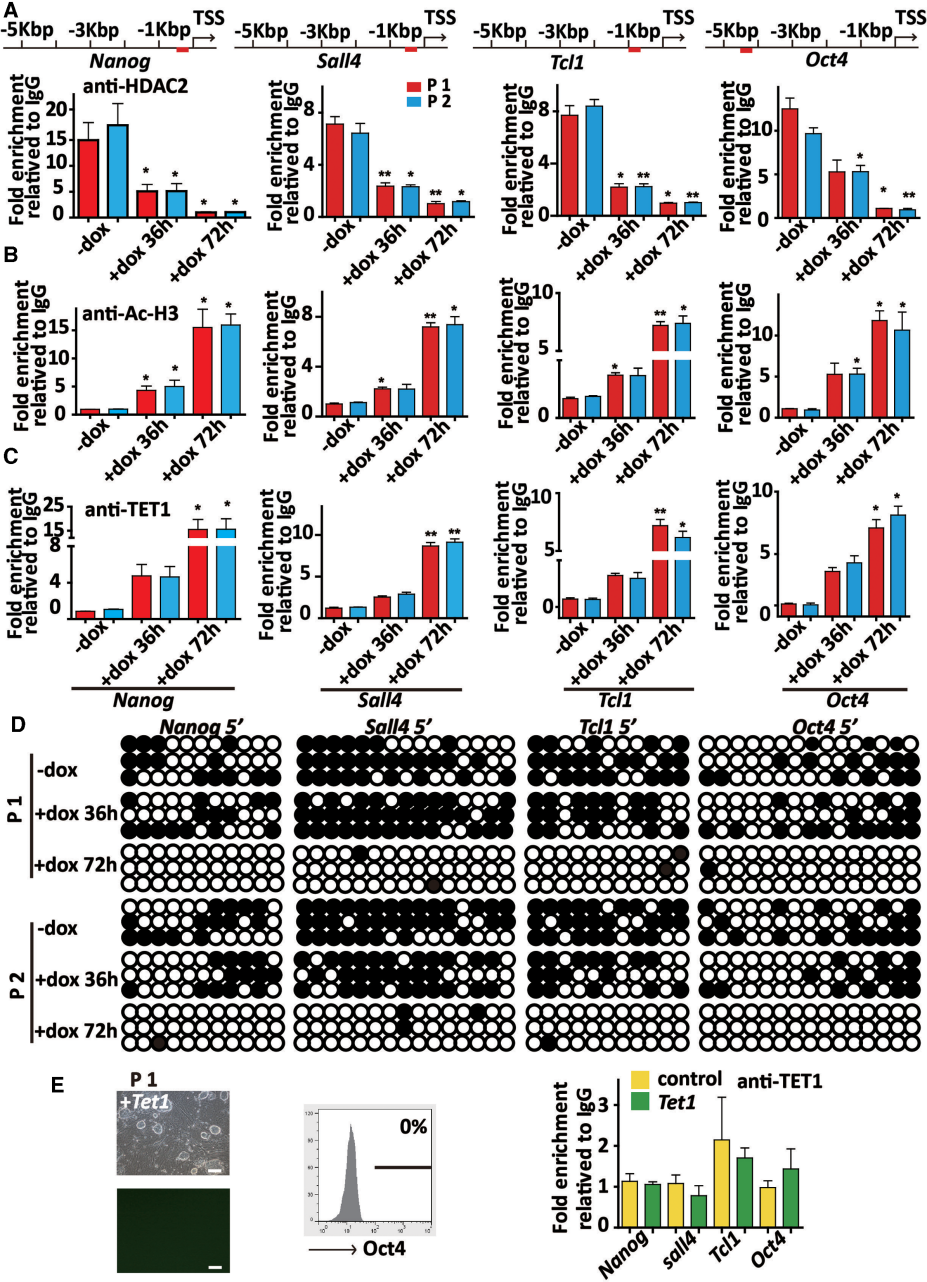
Histone acetylation is primarily required for DNA demethylation and maturation phase-related gene activation in the maturation of iPS cells. (**A**) ChIP-qPCR analysis of HDAC2 binding to the *Nanog*, *Sall4, Tcl1* and *Oct4* promoters in P1 and P2 clones before and after dox treatment for 36 and 72 h. The fold enrichment relative to IgG controls is shown. (**B**) ChIP-qPCR analysis of the level of Ac-H3 binding to the *Nanog*, *Sall4, Tcl1* and *Oct4* promoters in P1 and P2 clones before and after dox treatment at 36 and 72 h. (**C**) ChIP-qPCR assay showing the binding of TET1 to the *Nanog*, *Sall4, Tcl1* and *Oct4* promoters in P1 and P2 clones before and after dox treatment for 36 and 72 h. (**D**) Bisulfite sequencing of the *Nanog*, *Sall4, Tcl1* and *Oct4* promoters in P1 and P2 clones before and after dox treatment for 36 and 72 h. (**E**) Phase and Oct4-GFP images of P1 clone transfected with *Tet1* (left). Scale bar, 100 μm. Flow cytometry analysis of Oct4-GFP reporter activity (middle). ChIP-qPCR analysis of the binding of TET1 to the *Nanog*, *Sall4, Tcl1* and *Oct4* promoters in P1 clone transfected with or without *Tet1* (right). The data in A, B, C and E represent the means ± S. E. M. of three independent experiments. *P < 0.05, **P < 0.01 (two-tailed Student's *t*-test).

To explore the molecular mechanism of the *Hdac2* knockdown-mediated activation of maturation phase-related genes, we examined the dynamics of the relative binding of HDAC2 and the level of histone acetylation in the promoter regions of the *Nanog*, *Sall4, Tcl1* and *Oct4* genes in P1 and P2 (two dox-inducible *shHdac2* pre-iPS cell clones) at different time after dox treatment. Chromatin immunoprecipitation quantitative PCR (ChIP-qPCR) data showed that HDAC2 accumulation gradually decreased, whereas histone acetylation (acetyl-histone H3, Ac-H3) significantly increased at these promoters following dox treatment (Figure 4a and b). Recent studies have shown that TET-mediated DNA demethylation at pluripotency gene promoters play an important role in reprogramming (27,31). Firstly, the expression of TET1 in P1 and P2 clones upon dox treatment was upregulated (Supplementary Figure S7a and b). Next, the binding of TET1 to the *Nanog*, *Sall4, Tcl1* and *Oct4* promoters in P1 and P2 clones increased at these regions after different lengths of dox treatment (Figure 4c), which coincided with an increase in histone acetylation. We also identified a reprogramming-related, differentially methylated region (R-DMR) in these genes that underwent DNA demethylation in P1 and P2 clones after dox treatment (Figure 4d). Interestingly, in the absence of *Hdac2* knockdown, the overexpression of *Tet1* (Supplementary Figure S7c and d) alone could not convert pre-iPS cells into mature iPS cells (Figure 4e) and the histone acetylation level at the examined gene promoters was also not affected in these cells (Supplementary Figure S7e), which is consistent with the published study (31). Further, the binding of TET1 at these regions did not increase after *Tet1* transfection (Figure 4e). These results suggest that the binding efficiency was the main factor for TET1 binding at the maturation phase-related gene promoters to promote the pre-iPS cell maturation.

### TET1-mediated DNA demethylation is critically involved in *Hdac2* knockdown-induced iPS cell maturation

**Figure 5.**
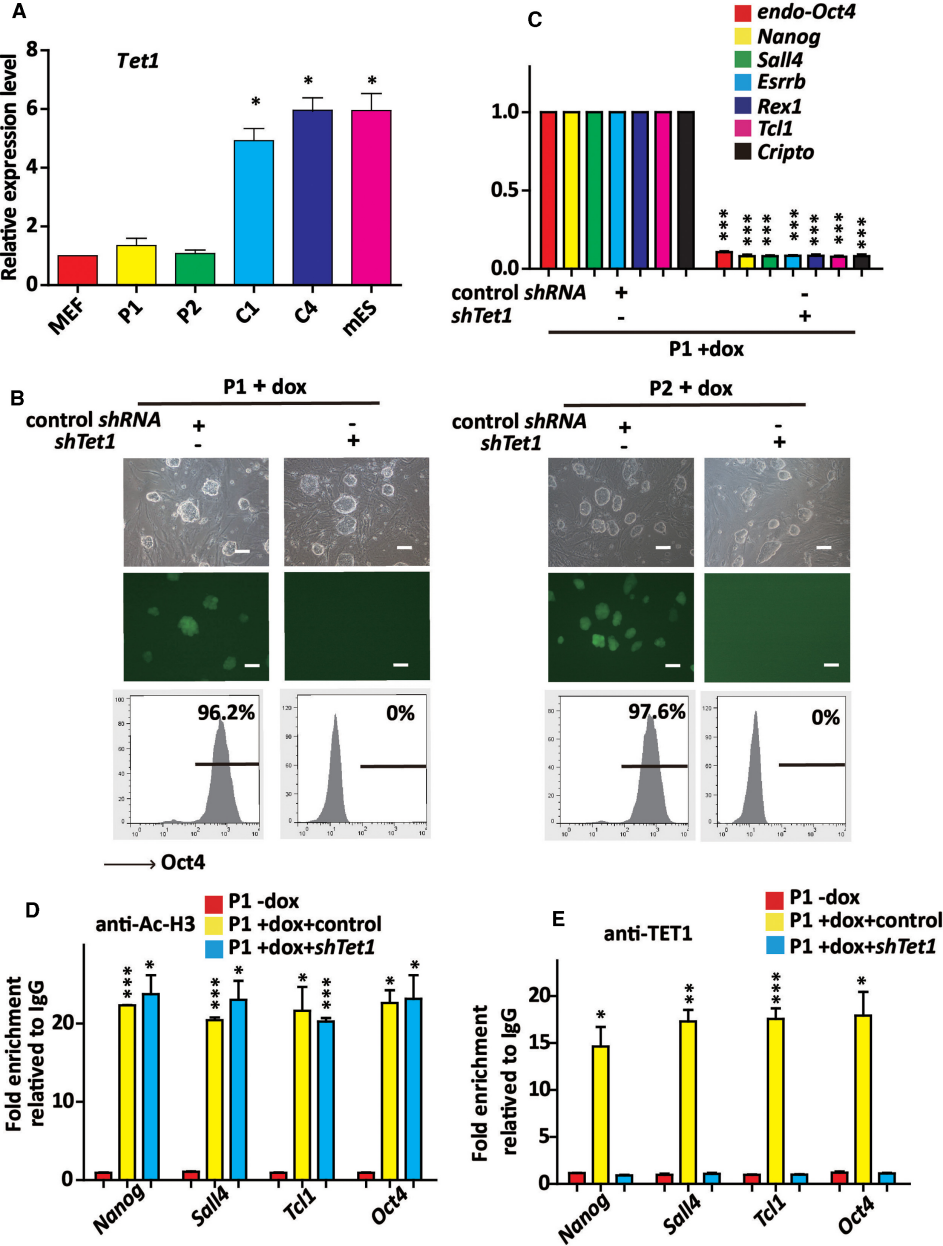
TET1-mediated DNA demethylation is critically involved in *Hdac2* knockdown-induced iPS cell maturation. (**A**) qTR-PCR analysis of *Tet1* expression in MEFs, P1, P2, C1, C4 and mES cells. *Actin* was used as an internal control. (**B**) Phase and Oct4-GFP images of P1 and P2 clones transfected with control shRNA and *shTet1* under dox treatment (upper). Scale bar, 100 μm. Flow cytometry analysis of the proportion of Oct4-GFP cells in P1 and P2 clones transfected with control shRNA and *shTet1* under dox treatment (below). (**C**) qRT-PCR analysis of maturation phase genes in P1 clone transfected with control shRNA and *shTet1* upon dox treatment. The mRNA levels normalized to *Actin* are relatived to that of the control (the P1 clone transfected with control shRNA under dox treatment). (**D**) The ChIP-qPCR assay showed the level of Ac-H3 to the *Nanog*, *Sall4, Tcl1* and *Oct4* promoters in P1 clone transfected with control shRNA, *shTet1* under dox treatment for 72 h. The fold enrichment relative to IgG controls is shown. (**E**) The ChIP-qPCR assay showed the binding of TET1 to the *Nanog*, *Sall4, Tcl1* and *Oct4* promoters in P1 clone transfected with control shRNA, *shTet1* under dox treatment for 72 h. The fold enrichment relative to IgG controls is shown. The data in A, C, D and E represent the means ± S. E. M. of three independent experiments. *P < 0.05, **P < 0.01, ***P < 0.001 (two-tailed Student's *t*-test).

Previous study showed that TET1 mediated changes in DNA methylation and hydroxymethylation play important roles in genome-wide epigenetic remodeling during reprogramming (27). We found that the expression of *Tet1* was higher in C1 and C4 (the converted iPS cells) and mES cells than in P1 and P2 clones by qRT-PCR (Figure 5a). Hence, we constructed lentiviral vector expressing shRNA which specifically targeted mouse *Tet1*. The efficiency of knockdown by the individual viruses in mES cells was confirmed by using qRT-PCR (Supplementary Figure S7f). When *Tet1* was knocked down, the P1 and P2 clones could not be efficiently converted into iPS cells upon dox treatment (Figure 5b). We also found that the expression of the maturation phase-related genes was not activated when *Tet1* was knocked down in P1 and P2 clones (Figure 5c and Supplementary Figure S8a) upon dox treatment. The increased Ac-H3 at the *Nanog*, *Sall4, Tcl1* and *Oct4* promoters caused by *Hdac2* knockdown were not affected by *Tet1* knockdown (Figure 5d and Supplementary Figure S8b), while the accumulation of TET1 at these promoters was decreased (Figure 5e and Supplementary Figure S8c). Combined with the previous study that TET1 and Nanog co-occupy genomic loci of genes associated with maintenance of pluripotency in embryonic stem cells and TET1 binding is reduced upon *Nanog* depletion (31), our data indicate that TET1 is required for *Nanog* activation and the binding of Nanog at its own and other maturation-related gene promoters might be also required for the efficient accumulation of TET1 binding at these regions. This speculation was supported by the finding that the *Nanog* knockdown significantly inhibited the demethylation of the *Nanog*, *Sall4*, *Tcl1* and *Oct4* promoters in dox treated P1 clone before or after *shNanog* transfection (Supplementary Figure S8d). Collectively, these results prove that the HDAC2-TET1 binding to histone-binding proteins at the maturation gene promoters played a critical role in transforming pre-iPS cells into fully reprogrammed iPS cells.

### An HDAC2-TET1 switch is a crucial modulator of maturation phase-related gene activation and iPS cell maturation

**Figure 6.**
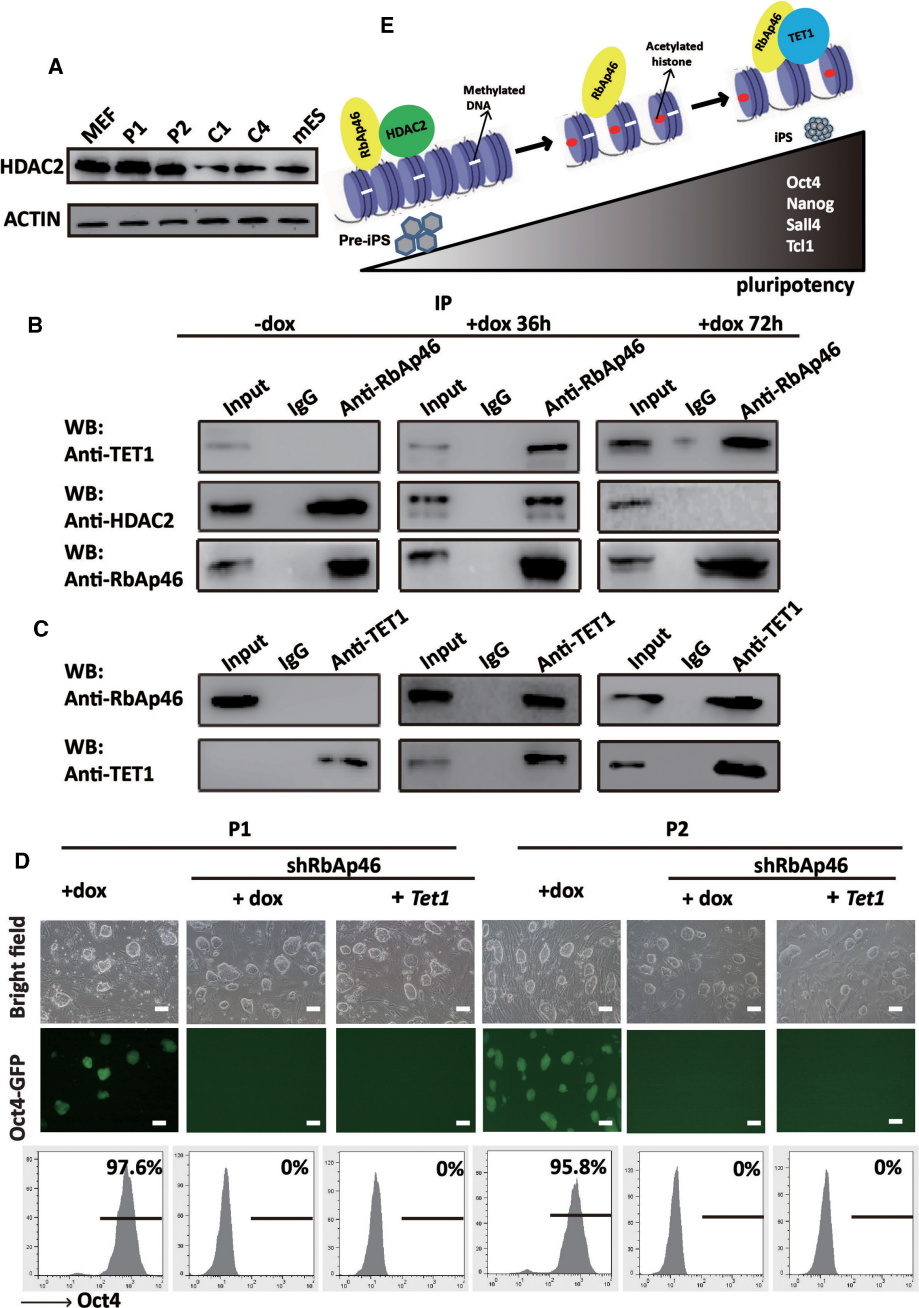
TET1 competes with HDAC2 for binding to RbAp46 in the further reprogramming of pre-iPS cells. (**A**) Representative western blot for HDAC2 in MEFs, P1, P2, C1, C4 and mES cells. ACTIN was used as an internal control. (**B**) The interaction between HDAC2, TET1 and RbAp46 in P1 clone before and after dox treatment for 36 and 72 h. Co-IP was performed using IgG or an RbAp46 antibody, followed by a western blot analysis of TET1, HDAC2 and RbAp46. (**C**) The reciprocal IP experiment showed that RbAp46 was found in the TET1 precipitate in P1 clone after dox treatment for 36 and 72 h. (**D**) Phase and Oct4-GFP images of P1 and P2 clones transfected with *shRbAP46* + *Tet1* or *shRbAp* under dox treatment or dox treatment alone. Scale bar, 100 μm. The results of a flow cytometry analysis of the proportion of Oct4-GFP cells are shown at the bottom. (**E**) A working model of competitive binding between HDAC2 and TET1 to the promoters of maturation phase-related gene during reprogramming.

We found that the expression of HDAC2 was higher in OG-MEFs, P1 and P2 cells than in the C1, C4 and mES cells (Figure 6a). Previous studies have shown that HDAC1, HDAC2 and the histone-binding proteins RbAp48 and RbAp46 form a core complex shared between the NuRD and Sin3-histone deacetylase complexes (32). In our study, an interaction between HDAC2 and RbAp46 was detected, which decreased during treatment with dox in P1 and P2 clones (Figure 6b and Supplementary Figure S9a). An interaction between TET1 and RbAp46 was also detected, which increased during treatment with dox in P1 and P2 clones (Figure 6c and Supplementary Figure S9b), indicating that RbAp46 interacts with different epigenetic modulators (HDAC2 or TET1) to perform different functions during iPS cell maturation. When *RbAp46* was knocked down in P1 and P2 clones, no Oct4-GFP expression was observed after dox treatment. Further, the overexpression of *Tet1* did not convert pre-iPS cells into mature iPS cells when *RbAp46* was knocked down (Figure 6d). These data show that an RbAp46-mediated HDAC2-TET1 switch is involved in the regulation of endogenous maturation phase-related gene expression and in the process of pre-iPS cells achieving full pluripotency.

## DISCUSSION

To completely reset the epigenome of a differentiated cell to a state of pluripotency, reprogramming factors must cooperate with chromatin-remodeling complexes to activate pluripotency-associated genes and to inactivate developmental genes (33,34). Studies have revealed that both DNA methyltransferase and HDAC inhibitors improve reprogramming (18). However, the molecular mechanisms of the interplay between histone acetylation and active DNA demethylation in cell reprogramming remain unclear. In the present study, we found evidence of a negative role for HDAC2 in the induction of pluripotency by the OSKM reprogramming system. The knockdown of endogenous *Hdac2* facilitated iPS cell maturation by activating maturation-related genes. Moreover, we revealed that HDAC2 competed with TET1 to binding of RbAp46 at maturation-related gene promoters to impede the activity of maturation-related genes (Figure 6e).

Reprogramming is divided into initiation, maturation and stabilization phases (3). The initiation phase involves a MET (mesenchymal to epithelial transition), which is characterized by the upregulation of epithelial genes, morphological transformation into epithelial-like colonies and the appearance of AP- and SSEA1-positive cells. The MET was found to be an important process for the initiation phase, and some factors, such as E-cadherin and miR-200, promoted iPS generation by accelerating the MET (4,7–8,35).The maturation phase is characterized by the high expression of *Nanog*, *Sall4* and *Tcl1*. The activation of these maturation phase-related genes is essential for achieving pluripotency (3). Selection or screening for the activation of endogenous *Nanog* expression facilitates the isolation of fully reprogrammed iPS cells. Furthermore, Nanog promotes the transfer of pluripotency after ES cell fusion (15). Nanog may orchestrate the transition to the pluripotent ground state by facilitating the cooperative binding of core pluripotency factors to their cognate ES cell targets. Sall4 interacts with Nanog to form a positive regulatory loop for *Sall4* and *Nanog* (10). Mouse ES cells lacking Sall4 exhibited reduced proliferation ability (36). The overexpression of *Sall4* to the reprogramming factors increased the number of iPS colonies generated from MEFs (37). The knockdown or knockout of *Tcl1* in mES cells impaired self-renewal by inducing differentiation and repressing proliferation (38–40). However, the mechanism underlying the maturation of pre-iPS cells was largely unknown. Our results suggest that HDAC2, a class I HDAC member, may control a rate-limiting step in reprogramming. We found that *Hdac2* knockdown specifically promoted the expression of maturation phase-related genes compared with that of genes involved in other phases. The knockdown of *Hdac2* further promoted the transition of iPS cells from a pre-maturation state to a fully pluripotent state and was associated with increases in the expression of the maturation-related genes and the differentiation potential of iPS cells. Together with the critical role of Nanog as a key pluripotency factor that activates maturation-related genes; our study found that the transition induced by the knockdown of *Hdac2* could be blocked by the knockdown of *Nanog*. In conclusion, the present study revealed that HDAC2 is an intrinsic negative regulator of the maturation of iPS cells, which significantly suppresses iPS cell maturation by preventing the activation and expression of maturation-related genes.

Previous studies have revealed that the overexpression of both TET and Nanog synergetically increased reprogramming efficiency, whereas silencing TET1 expression led to reduced reprogramming efficiency. Moreover, TET1 was able to replace Oct4 to generate fully pluripotent iPS cells, which was partly achieved by promoting endogenous *Oct4* gene demethylation. Recently, it was shown that Tet-deficient MEFs could not be reprogrammed due to the prevention of the MET step (41). Based on our data, TET1 play a very important role in the maturation of iPS cells by catalyzing the demethylation of DNA in the promoter regions of maturation phase-related genes.

After the initiation of somatic cell reprogramming, large numbers of previously expressed genes have been shown to be silenced, whereas *endo*-*Oct4* and other pluripotency genes were sequentially activated and expressed in the cells (5). It was shown that histone acetylation at distinct gene regions is the first step in the switch between active and repressive chromatin status, and DNA methylation patterns may also guide the final pattern of histone modifications (including histone deacetylation and methylation) in silent gene regions (42). The molecular interplay between histone acetylation and DNA demethylation in the activation of pluripotency genes remains largely unclear. In the present study, we demonstrated that there is a primary requirement for histone acetylation than for DNA demethylation to the activation of pluripotency genes for iPS cell maturation. Our results showed that HDAC2 bound to maturation phase-related gene promoters in pre-iPS cells, whereas the same promoter region was occupied by TET1 in fully reprogrammed iPS cells. Moreover, in the transition of pre-iPS cells to iPS cells induced by *Hdac2* knockdown, the levels of histone acetylation and binding of TET1 to these promoters were significantly increased. Importantly, the overexpression of *Tet1* did not trigger the transcriptional activity of these genes under the condition of histone hypoacetylation at their promoters. Furthermore, we found that HDAC2 competed with TET1 for binding to these promoter regions. Thus, we concluded that the knockdown of *Hdac2* in pre-iPS cells allows the direct binding of TET1 to maturation phase-related gene promoters, thereby activating these genes.

Previous studies have shown that HDAC2 and histone-binding proteins (such as RbAp48 and RbAp46) form a core complex shared by the NuRD (43) and Sin3-histone deacetylase complexes (44). An analysis of the NuRD subunits revealed a histone deacetylase core complex and a connection with DNA methylation (32). Here, we showed that RbAp46, a histone-binding protein, interacted with HDAC2 in pre-iPS cells, and the elimination of HDAC2 facilitated the interaction of RbAp46 with TET1, which promoted the transition of iPS cells from an immature state to a mature state (45). Consistent with this observation, the knockdown of *RbAp46* blocked the *Hdac2* knockdown-induced transition of pre-iPS cells. These data suggest that the histone-binding protein RbAp46 interacts with different epigenetic modulators, either HDAC2 or TET1, to exert different functions in reprogramming. Collectively, our results revealed that during iPS cell maturation, histone acetylation was pre-required and an RbAp46-mediated HDAC2-TET1 switch served as a novel epigenetic regulator in the promoter regions of maturation genes.

Our data demonstrate not only that HDAC2 specifically impedes the maturation of somatic cell reprogramming but also that HDAC2 binds directly to the promoters of maturation phase-related genes and blocks their activation during iPS cell maturation. Our study results define the roles and mechanisms of an HDAC2-TET1 switch in cell reprogramming, thus not only improving our understanding of cell reprogramming and the interplay between histone acetylation and DNA demethylation in gene regulation but also providing guidance for the more efficient generation of high-quality, fully reprogrammed iPS cells.

## SUPPLEMENTARY DATA

Supplementary Data are available at NAR Online.

SUPPLEMENTARY DATA
