# Supplementary material for: An HDAC2-TET1 switch at distinct chromatin regions significantly promotes the maturation of pre-iPS to iPS cells
Source: Nucleic Acids Res. 2015 May 1;43(11):5409–22. doi: 10.1093/nar/gkv430 (PMC4477660; doi:10.1093/nar/gkv430)
Supplement: SUPPLEMENTARY DATA [file supp_43_11_5409__index.html]

An HDAC2-TET1 switch at distinct chromatin regions significantly promotes the maturation of pre-iPS to iPS cells — An HDAC2-TET1 switch at distinct chromatin regions significantly promotes the maturation of pre-iPS to iPS cells — SUPPLEMENTARY DATA 

# An HDAC2-TET1 switch at distinct chromatin regions significantly promotes the maturation of pre-iPS to iPS cells

## SUPPLEMENTARY DATA

**Files in this Data Supplement:**

- SUPPLEMENTARY DATA
