# Supplementary material for: An HDAC2-TET1 switch at distinct chromatin regions significantly promotes the maturation of pre-iPS to iPS cells
Source: Nucleic Acids Res. 2015 May 1;43(11):5409–22. doi: 10.1093/nar/gkv430 (PMC4477660; doi:10.1093/nar/gkv430)
Supplement: SUPPLEMENTARY DATA [file supp_gkv430_nar-00324-v-2015-File008.doc]

1. **SUPPLEMENTARY FIGURES**

**
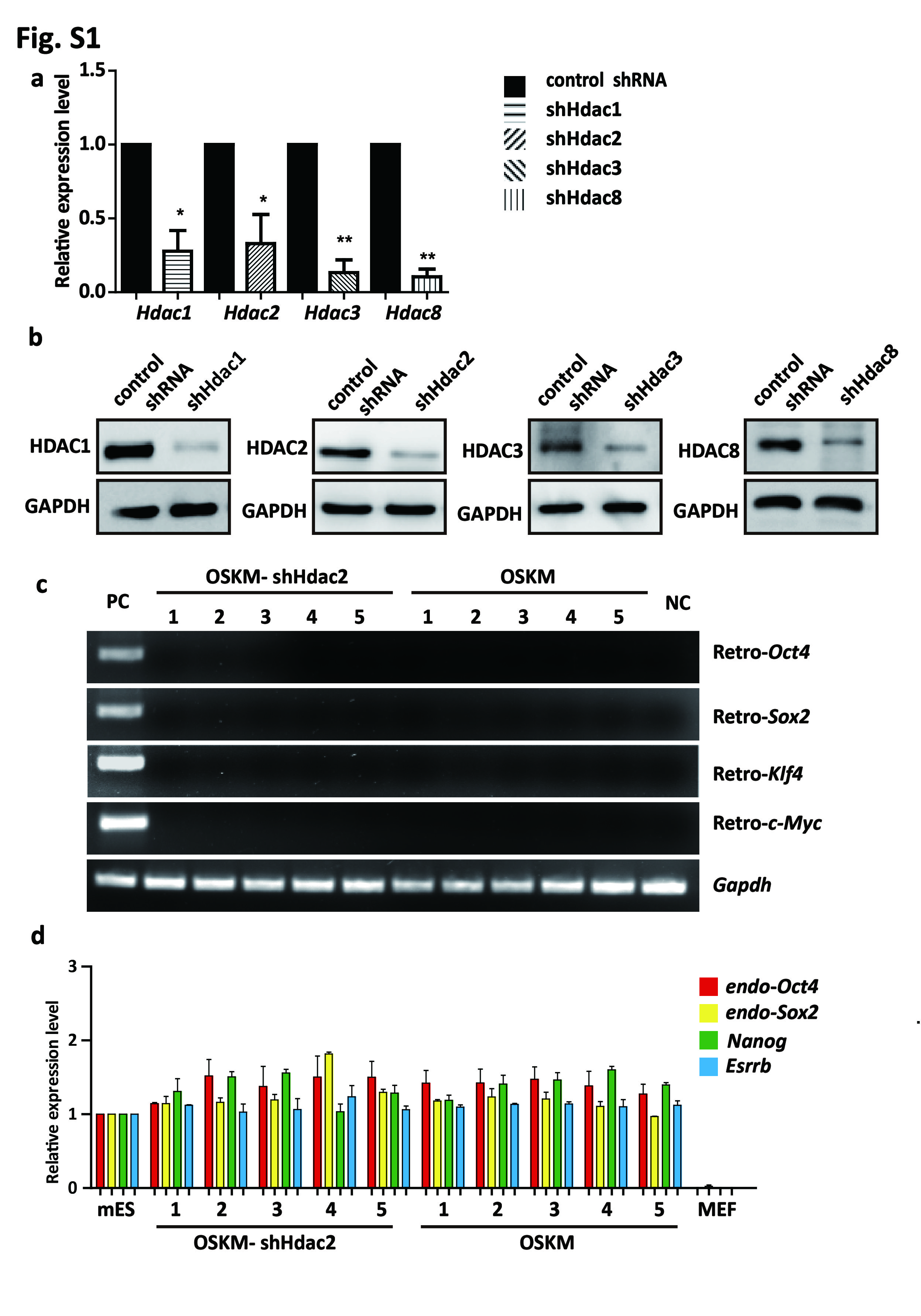
**

**Figure S1, Related to Figure 1. *Hdac2* knockdown did not sacrificed the pluripotency and differentiation of iPS Cells.** (a) qRT-PCR analysis of the targeting genes (*Hdac1*, *Hdac2*, *Hdac3* and *Hdac8*) in shRNA-treated MEFs. The mRNA levels were relatived to MEFs infected with control shRNA. *Actin* was used as the internal control. (b) Representative western blots for HDAC1, HDAC2, HDAC3 and HDAC8 protein expression in shRNA-treated MEFs respectively. GAPDH was used as the internal control. (c) The silence of exogenous genes was determined by RT-PCR at second passage of clones. OG-MEFs were used as a negative control (NC) and OG-MEFs infected with OSKM viruses after 48h as positive controls (PC). (d) qRT-PCR analyses of pluripotency markers (*endo-Oct4*, *endo-Sox2*, *Nanog* and *Essrb*) in the OSKM-iPS cell clones, the OSKM-shHDAC2-iPS cell clones, mES cells and MEFs. The mRNA levels normalized for *Actin* were relatived to mES cells. *P <0.05, **P <0.01 (two-tailed Student’s t-test).


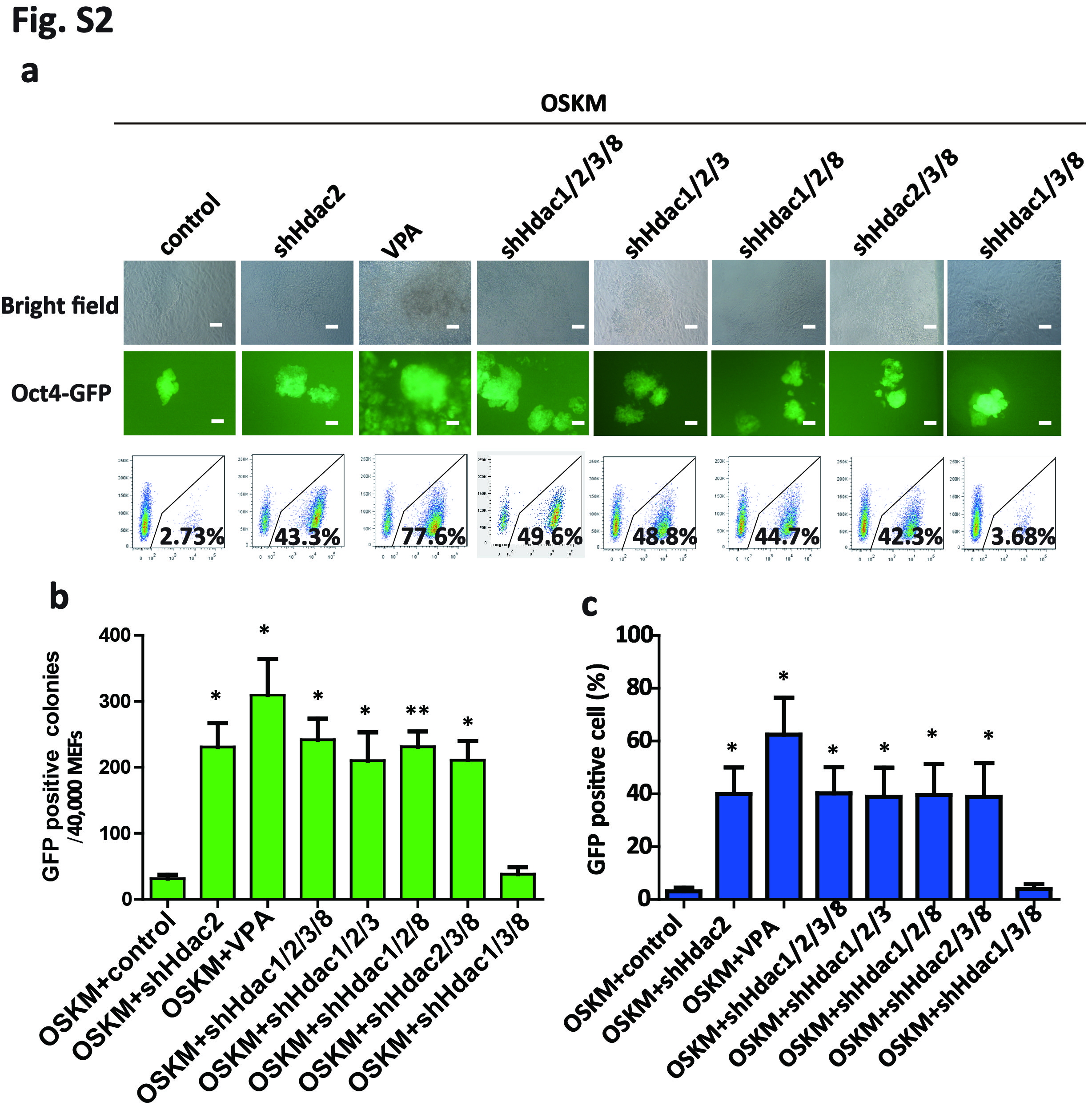


**Figure S2, Related to Figure 1. Hdac2 was the major factor involved in the repression of reprogramming.** (a) Morphologies of shHdacs or VPA iPS clones are observed at 12 day after OSKM viral transduction. FACS analyses of shHdacs or VPA reprogrammed Oct4-GFP MEFs at 12 day post-viral transduction were performed. Top: phase contrast; Middle: Oct4-GFP; Bottom: FACS data. Scale bars, 50 μm. (b) The number of Oct4-GFP positive colonies was measured at 12 day post-infection, and compared to OSKM–infected MEFs. (c) The percentage of Oct4-GFP positive cells was tested by FACS analysis at 12 day post-infection, and compared to OSKM–infected MEFs. *P <0.05, **P <0.01 (two-tailed Student’s t-test).


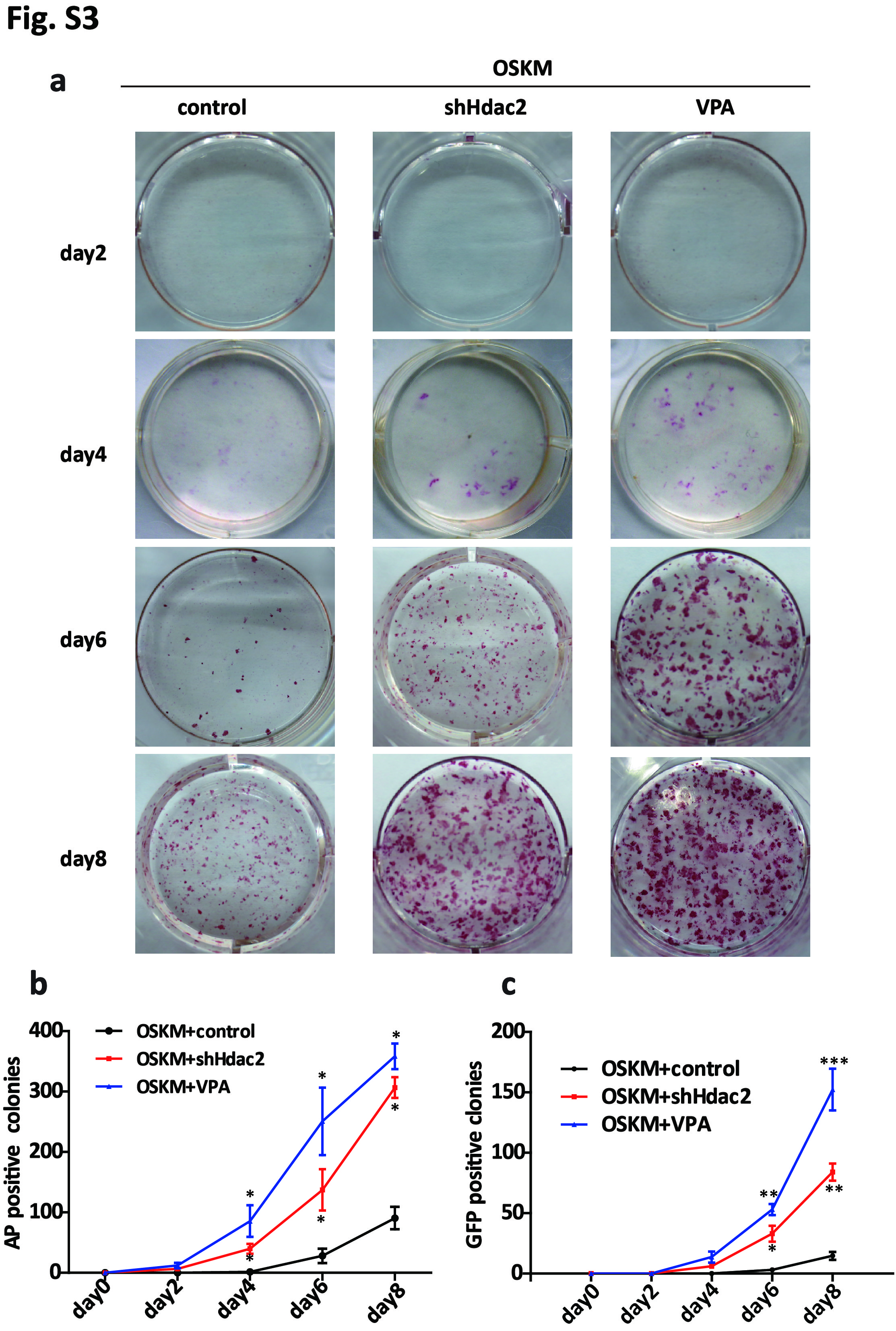


**Figure S3, Related to Figure 1. *Hdac2* knockdown or VPA treatment could accelerate the reprogramming.** (a) Representative images of AP positive colonies induced with shHdac2 or VPA treatment was captured at 2, 4, 6, 8 day after OSKM viruses infection. (b) The number of AP positive colonies induced with shHdac2 or VPA treatment was quantified at 2, 4, 6, 8 day after OSKM viruses infection. (c) The number of Oct4-GFP positive colonies induced with shHdac2 or VPA treatment was measured at 2, 4, 6, 8 day post-infection, and compared to OSKM–infected MEFs. *P <0.05, **P <0.01 ***P <0.001 (two-tailed Student’s t-test).


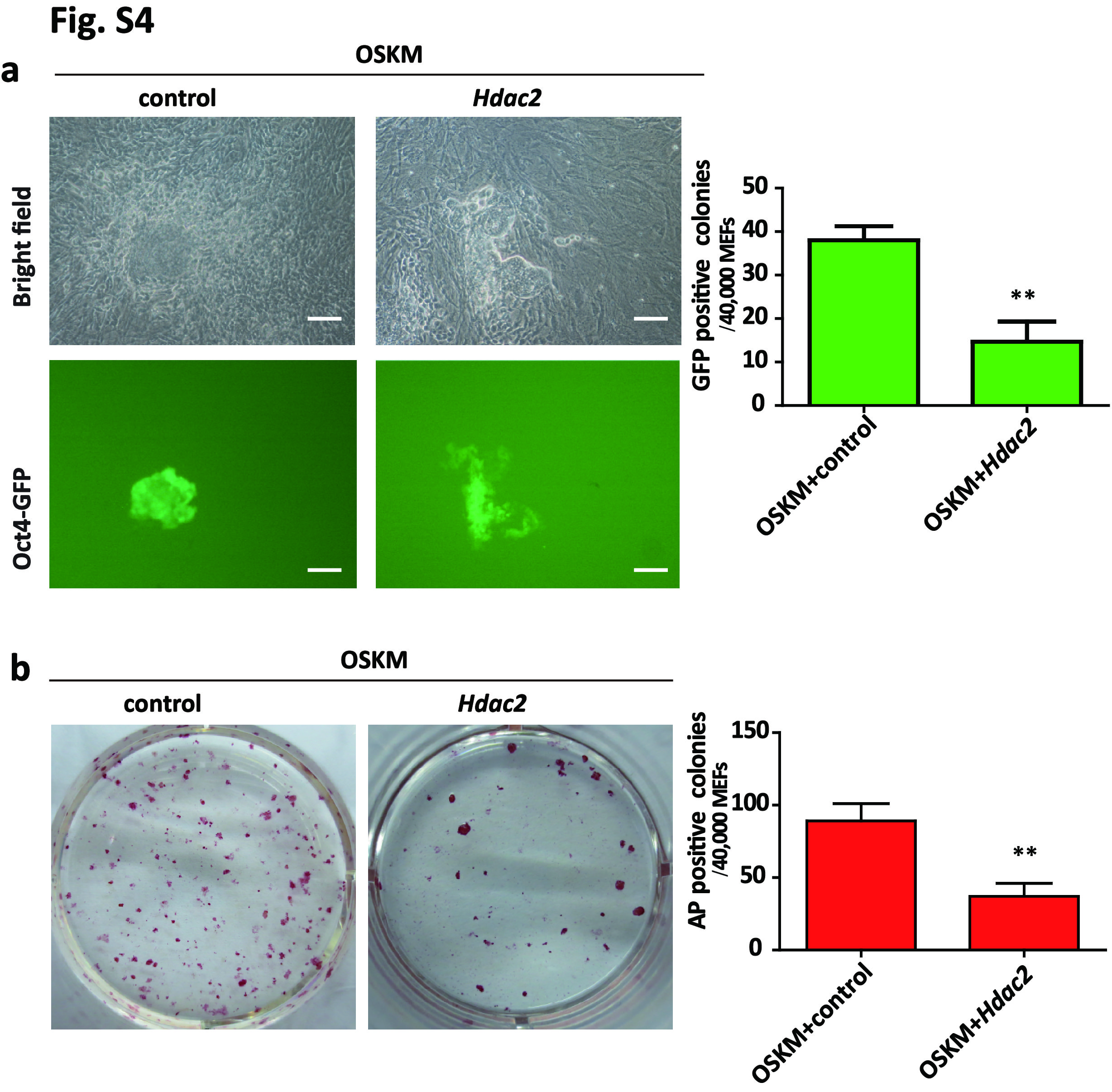


**Figure S4, Related to Figure 1. *Hdac2* overexpression inhibited the reprogramming.** (a) The number of Oct4-GFP positive colonies induced with *Hdac2* overexpression was measured at 12 day post-infection, and compared to OSKM–infected MEFs. Left: Morphology of iPS colonies; Right: Quantification. Scale bar, 100 μm. (b) The number of AP positive colonies induced with *Hdac2* overexpression was measured at 12 day postinfection, and compared to OSKM–infected MEFs. Left: Images of AP positive colonies; Right: Quantification. **P <0.01 (two-tailed Student’s t-test).


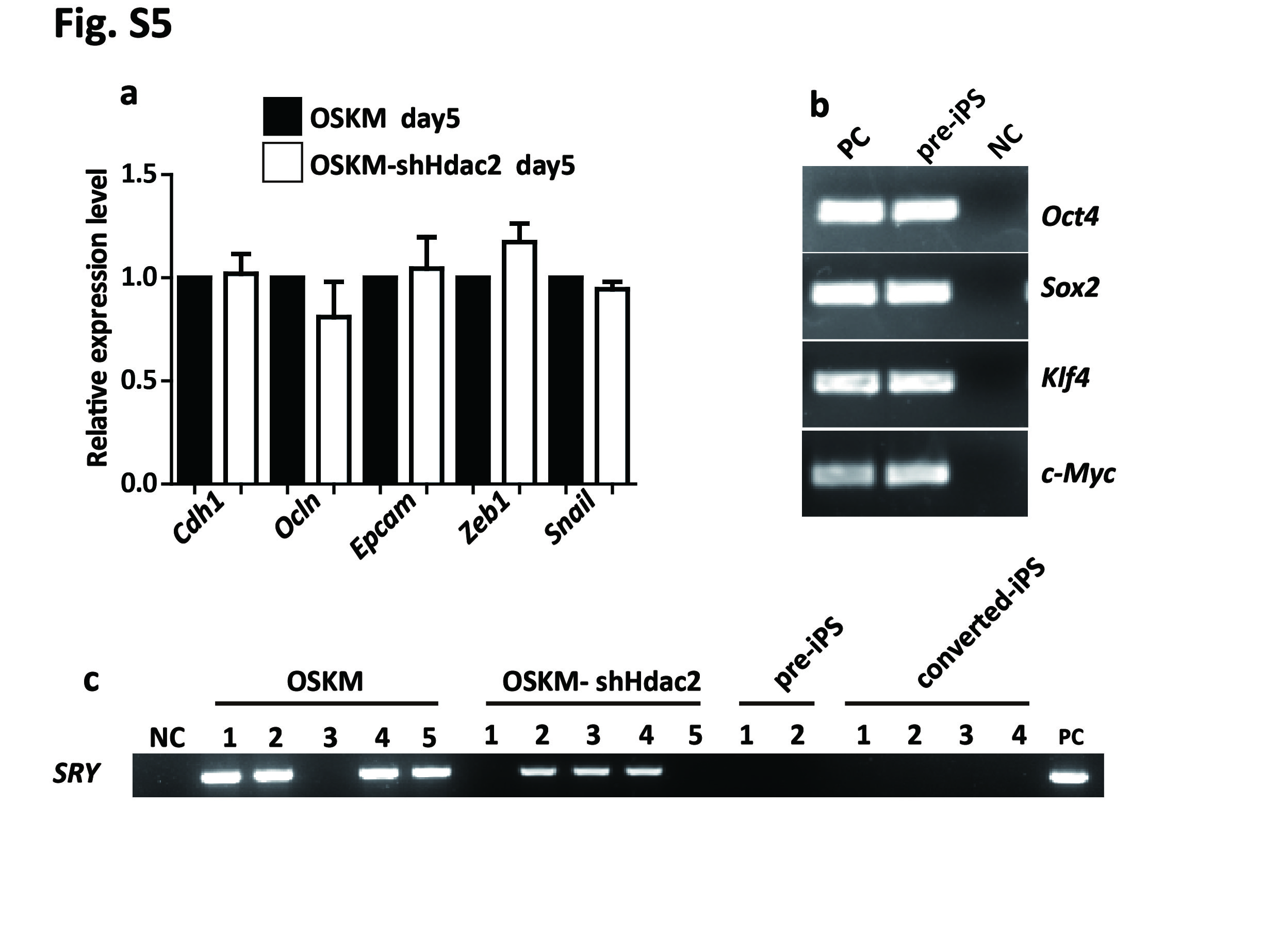


**Figure S5, Related to Figure2. The expression of the initiation phase-related genes did not significantly increase.** (a) qRT-PCR analyses of initiation phase-related genes (*Cdh1*, *Ocln*, *Epcam*, *Zeb1* and *Snail*) in MEFs infected with OSKM supplementation with or without *shHdac2* at day 5. (b) Genomic PCR analyses showed that the pre-iPS cells were infected with OSKM. OG-MEFs were used as a negative control (NC) and OG-MEFs infected with OSKM viruses after 48h as positive controls (PC). (c) The gender of the five conditional iPS cell lines, five OSKM-shHdac2 iPS cell lines, two pre-iPS cell lines and four converted iPS cell lines. *SRY*: sex-determining region of the chromosome Y.


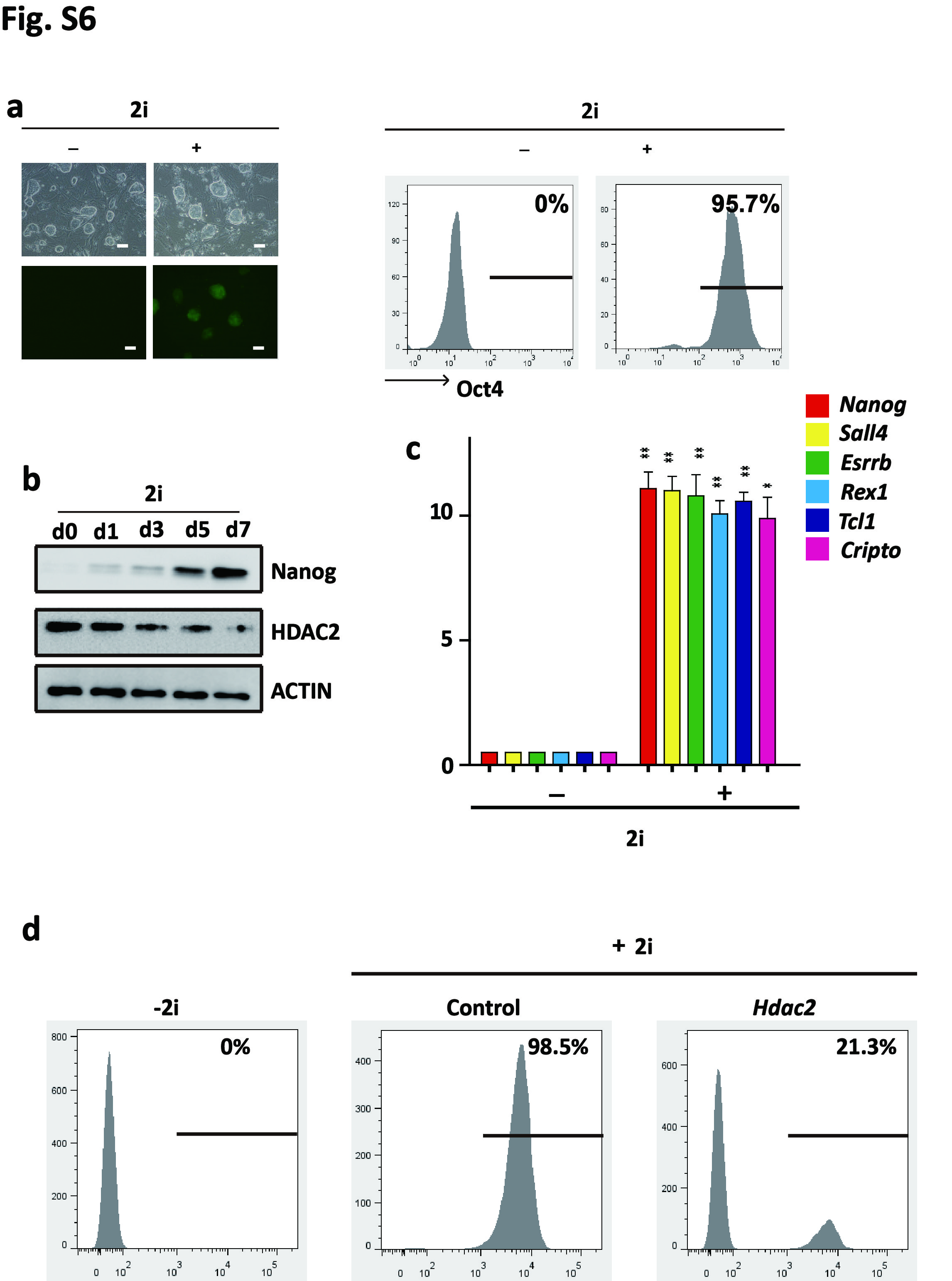


**Figure S6, Related to Figure 3. 2i/LIF was sufficient to promote the transition of pre-iPS cells to full pluripotency under serum-free conditions.** (a) Phase contrast and ﬂuorescence images of P1 clone treated with 2i/LIF in serum-free condition for 7 days. Scale bar, 100 μm. Flow cytometry analysis of Oct4-GFP reporter activity (right). (b) Western blot analysis for HDAC2 and Nanog expression in P1 clone treated with 2i/LIF under serum-free condition. (c) qRT-PCR analysis of maturation phase-related genes (*Nanog*, *Sall4*, *Esrrb*, *Rex1*, *Tcl1* and *Cripto*) in P1 clone treated as in (a). *Acitin* was used as an internal control. (d) Flow cytometry analysis of Oct4-GFP reporter activity in P1 clone upon 2i/LIF treatment before or after *Hdac2* transfection, and compared to P1 clone–treated with no chemicals. *P <0.05, **P <0.01 (two-tailed Student’s t-test).


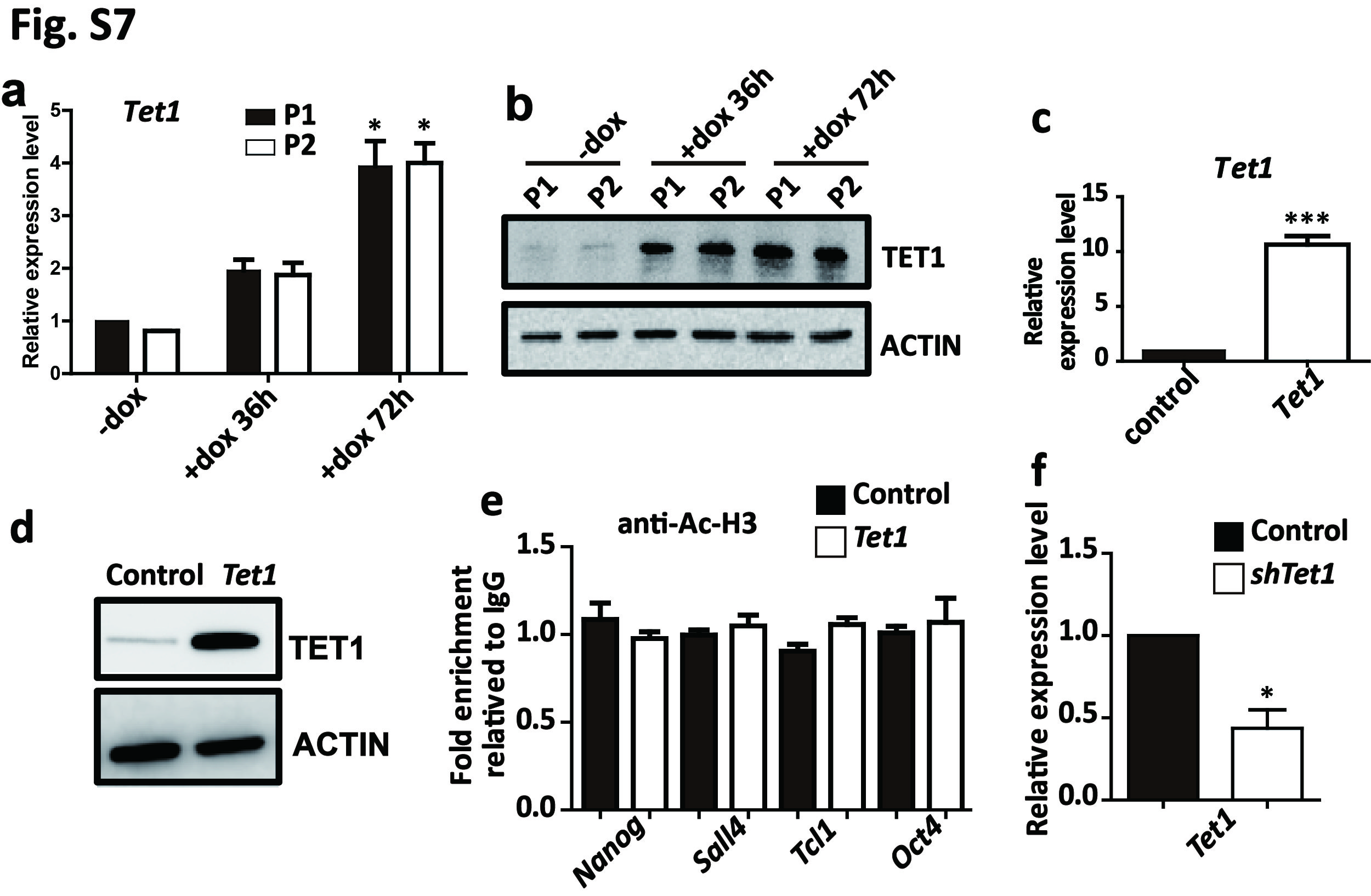


**Figure S7, Related to Figure 4. The histone acetylation did not increase at the maturation phase-related gene promoters after *Tet1* overexpression.** (a and b) qRT-PCR (a) and western blot (b) analyses of TET1 expression upon dox treatment in the P1 and P2 clones. ACTIN was used as an internal control. (c and d) qRT-PCR (c) and western blot (d) analyses of TET1 expression in P1 clone transfected with control vector or *Tet1* at 72h. ACTIN was used as an internal control. (e) ChIP-qPCR analysis of the level of Ac-H3 binding to the *Nanog*, *Sall4*, *Tcl1* and *Oct4* promoters in pre-iPS cell clone 1 transfected with control vector or *Tet1* at 72h. (f) qRT-PCR analysis of *Tet1* expression in shRNA-treated mES cells. The mRNA levels were relatived to mES cells infected with control shRNA. *Actin* was used as the internal control. *P <0.05, ***P <0.001 (two-tailed Student’s t-test).


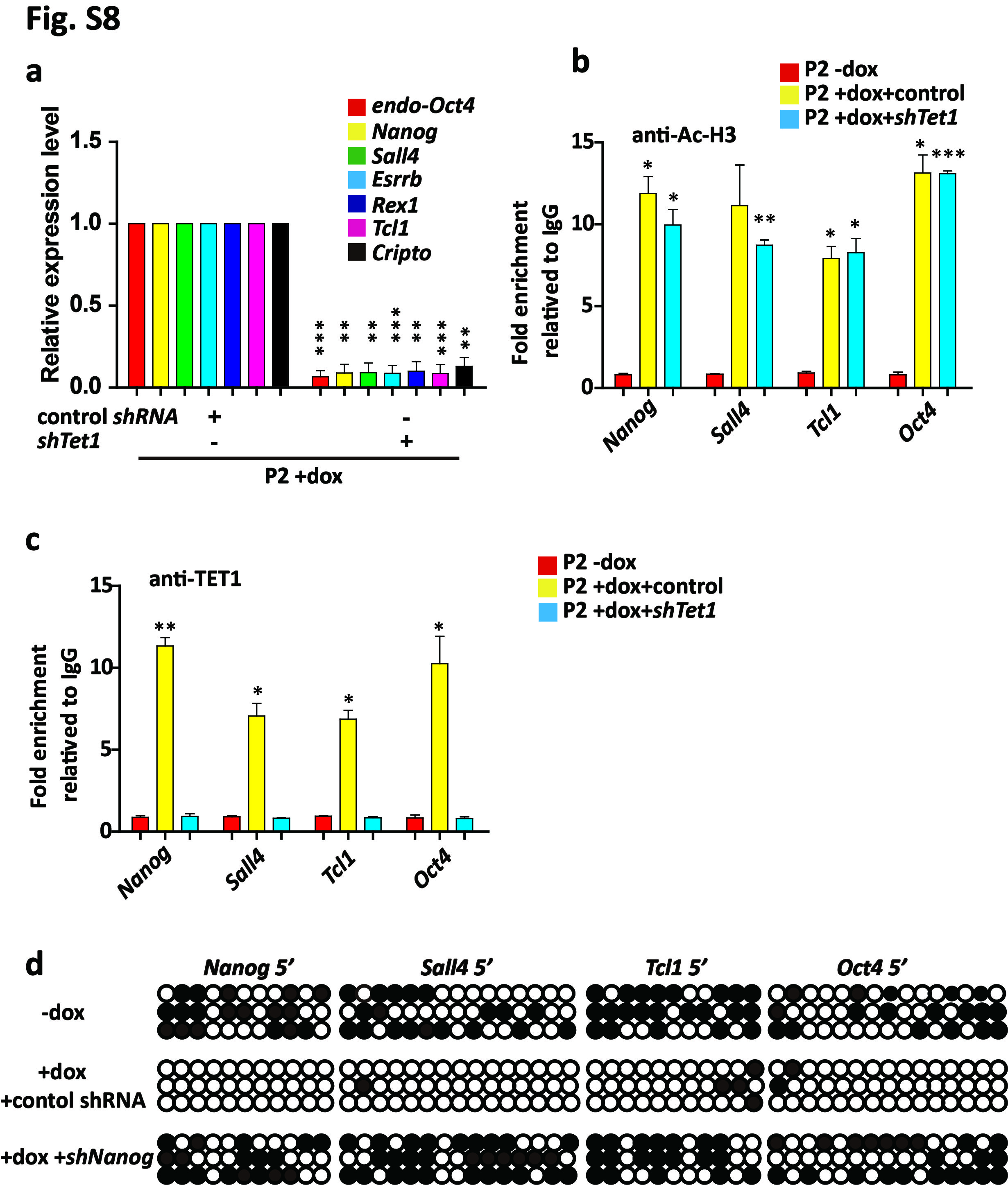


**Figure S8, Related to Figure 5. TET1-induced DNA demethylation acted downstream of HDAC2 in the pre-iPS cell maturation.** (a) qRT-PCR analysis of maturation phase-related gene expressions in P2 clone transfected with control shRNA or *shTet1* upon dox treatment. The mRNA levels normalized to *Actin* are relatived to that of the control (the P2 cell clone transfected with control shRNA under dox treatment). (b) The ChIP-qPCR assay showed the level of Ac-H3 to the *Nanog*, *Sall4, Tcl1* and *Oct4* promoters in P2 clone transfected with control shRNA or *shTet1* under dox treatment for 72h. The fold enrichment relative to IgG controls is shown. (c) The ChIP-qPCR assay showed the binding of TET1 to the *Nanog*, *Sall4, Tcl1* and *Oct4* promoters in P2 clone treated as in (b). The fold enrichment relative to IgG controls is shown. **(**d**)** Bisulfite sequencing of the *Nanog*, *Sall4,* *Tcl1* and *Oct4* promoters in pre-iPS cell clone 1 transfected with control shRNA or *shNanog* upon dox treatment. *P <0.05, **P <0.01, ***P <0.001 (two-tailed Student’s t-test).


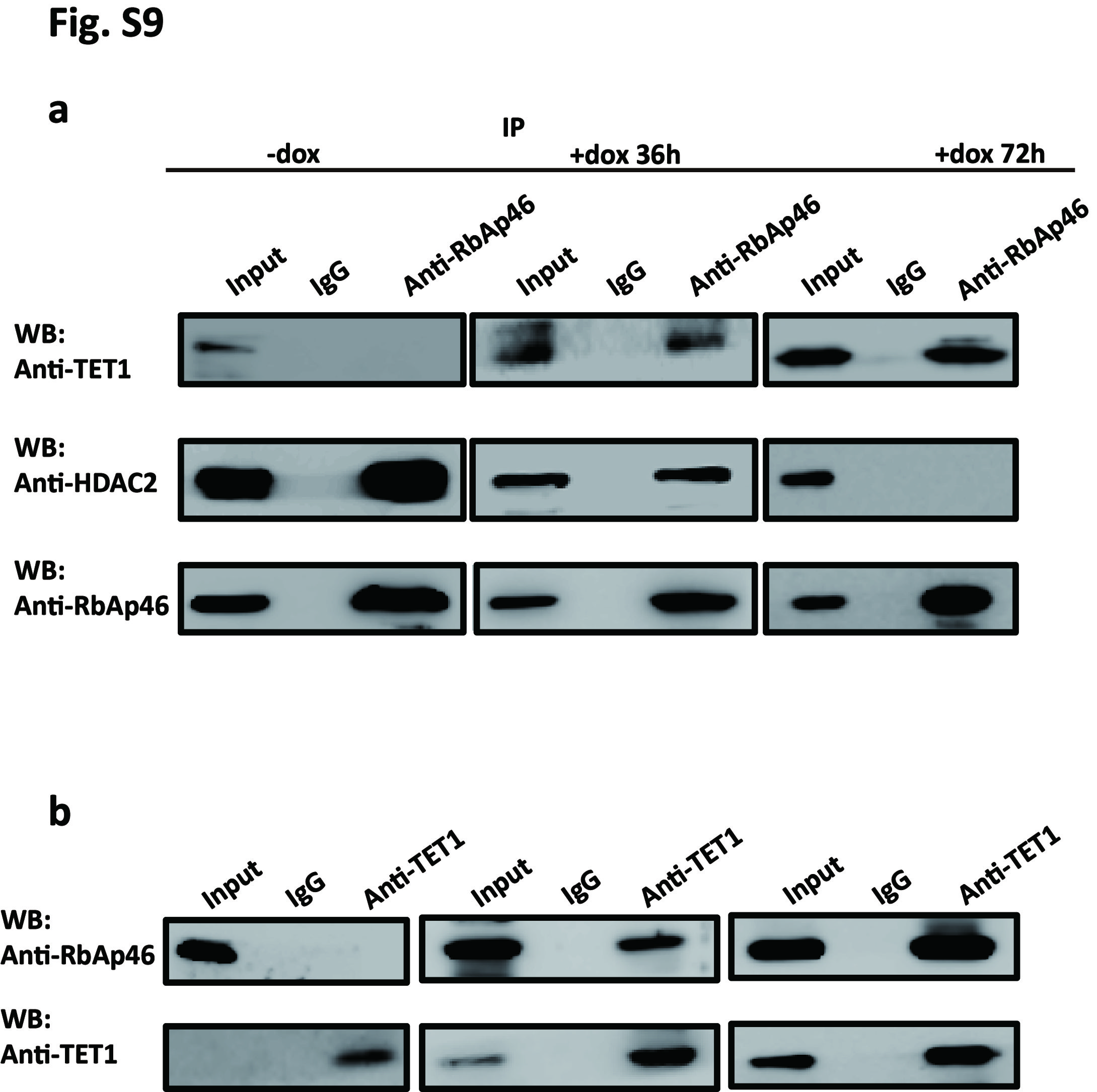


**Figure S9, Related to Figure 6. Co-IP analyses of the interaction of RbAp46 with HDAC2 or TET1 in P2 clone.** (a) The interaction between HDAC2, TET1 and RbAp46 in P2 clone before and after dox treatment for 36h and 72h. Co-IP was performed using IgG or an RbAp46 antibody, followed by a western blot analysis of TET1, HDAC2 and RbAp46. (b) The reciprocal IP experiment showed that RbAp46 was found in the TET1 precipitate in P2 clone after dox treatment for 36h and 72h.

**II. SUPPLEMENTARY METHODS**

**Vectors and shRNA**

Vectors expressing pMX-Oct4, Sox2, Klf4 and c-Myc were previously described . DNA fragments encoding shRNAs targeting *Hdac1*, *Hdac2*, *Hdac3* and *Hdac8* were cloned into retroviral vector pMKO.1. DNA fragments encoding shRNAs targeting *Tet1* and *RbAp46* were cloned into lentivirus vector pLKO.1. The shRNA sequences for *shHdac2-1* and *shHdac2-2*, which utilize the constitutive pLKO vector, were purchased as oligonucleotides and were cloned into pLKO-Tet-On. All the plasmids were verified via DNA sequencing. The shRNA for *Nanog* was commercially synthesized (Invitrogen). All the primers are described in Supplementary Table S1.

**Genomic PCR, RT-PCR and qRT-PCR**

Genomic DNA was extracted from iPS cells using a DNA extraction kit (TIANGEN). Total RNA was extracted from cells using the TRNzol-A+ total RNA Extraction Kit (TIANGEN). cDNA synthesis was performed with the TIANScript RT Kit (TIANGEN). The template for RT-PCR was equivalent to 20 ng of total RNA. Quantitative PCR was performed for 40 cycles (Stratagene Mx3000p), and the results were normalized to the β-actin gene. The primer sequences for the genomic PCR, exogenous transgene silencing and qRT-PCR are listed in Supplementary Table S1.

**Alkaline Phosphatase staining and Immunostaining**

AP staining was performed using the Fast Red Alkaline Phosphatase Kit (Sigma) according to the manufacturer's protocol. For immunostaining, briefly, cells were washed twice with PBS and fixed with 4% paraformaldehyde at room temperature for 20 min. The fixed cells were permeabilized with 0.2% Triton X-100 for 5 min and blocked in PBS containing 10% FBS for 1 h at room temperature. The primary antibody was diluted 1:1000 in PBS containing 10% FBS. The cells were incubated with the primary antibody overnight at 4°C and were then washed three times with PBS. The secondary antibody was diluted 1:1500, and the cells were incubated for 45 min in the dark at room temperature. The cells were stained with antibodies and counterstained with Hoechst33342. Finally, the cells were examined under a fluorescence microscope to obtain images. The antibodies used are listed in Supplementary Table S2.

**Western Blotting and co-Immunoprecipitation**

Equal amounts of cell lysates were separated on polyacrylamide-SDS gels, blotted on a nitrocellulose filter membrane and probed with the antibodies. The antibodies used are listed in Supplementary Table S2. GAPDH or actin was used as loading controls. After incubation with the appropriate secondary antibodies, signals were visualized using enhanced chemiluminescence (ECL, ImageQuant LAS 4000 mini).

Co-immunoprecipitation was performed as previously described. Cell lysates (400 μl for each immunoprecipitation) were immunoprecipitated with the appropriate antibodies (3 μg for each immunoprecipitation, listed in Supplementary Table S2), followed by western blotting. Mouse IgG or rabbit IgG antibody was used as a control.

**Promoter Methylation Analyses**

Promoter CpG methylation was analyzed using bisulfite PCR as described in our previous study . Briefly, genomic DNA was extracted using a genomic DNA extraction kit (TIANGEN). A 1 μg aliquot of DNA was then used for DNA methylation analysis with bisulfite PCR primers. The products were cloned into the pMD19-T vector (Takara) and sequenced. The bisulfite PCR primers are listed in Supplementary Table S1.

**Data Analysis**

The statistically analyzed data are presented as the means ± the standard error of the mean (S.E.M.) of three independent experiments, as indicated. P values were calculated using Student's two-tailed t-test. *P <0.05, **P <0.01, ***P <0.001 (two-tailed Student’s t-test).

**III. SUPPLEMENTAL REFERENCES**

1. Takahashi, K. and Yamanaka, S. (2006) Induction of pluripotent stem cells from mouse embryonic and adult fibroblast cultures by defined factors. *Cell*, **126**, 663-676.

2. Zhu, S., Wang, W., Clarke, D.C. and Liu, X. (2007) Activation of Mps1 Promotes Transforming Growth Factor-beta-independent Smad Signaling. *Journal of Biological Chemistry*, **282**, 18327-18338.

3. Guo, X., Liu, Q., Wang, G., Zhu, S., Gao, L., Hong, W., Chen, Y., Wu, M., Liu, H., Jiang, C. *et al.* (2013) microRNA-29b is a novel mediator of Sox2 function in the regulation of somatic cell reprogramming. *Cell Res*, **23**, 142-156.

**SUPPLEMENTAL TABLE S1**

| Primer | | Sequence (5' to 3') | | | | | | | Applications | |
| --- | --- | --- | --- | --- | --- | --- | --- | --- | --- | --- |
| control | | CCGGTCCTAAGGTTAAGTCGCCCNCGTTCGAGCGAGGGCGACTTAACCTTAGGTTTTTG | | | | | | | vector | |
| *shHdac1* | | CCGGAAGCAGCGTCTCTTTGAGAACCTCGAGGTTCTCAAAGAGACGCTGCTTTTTTTG | | | | | | | vector | |
| *shHdac2* | | CCGGGTATCATCAGAGAGTCTTATTCTCGAGAATAAGACTCTCTGATGATACTTTTTG | | | | | | | vector | |
| *shHdac3* | | CCGGAACCTCATCGCCTGGCATTGACTCG  AGTCAATGCCAGGCGATGAGGTTTTTTTG | | | | | | | vector | |
| *shHdac8* | | CCGGTGAGCCCCACCGAATCCAATTCTCGAG TTGGATTCGGTGGGGCTCATTTTTTTG | | | | | | | vector | |
| *shTet1* | | CCGGGCTCATGGAGACTAGGTTTGGCTCGAGCCAAACCTAGTCTCCATGAGCTTTTTG | | | | | | | vector | |
| *shRbAp46* | | CCGGGCGTGTCATCAACGAAGAGTACTCGAGTACTCTTCGTTGATGACACGCTTTTTG | | | | | | | vector | |
| *Nanog* shRNA | | GCCUAAAACCUUUUCAGAATT | | | | | | | siRNA | |
| Dox-inducible-*shHdac2-1* | | CCGGGTATCATCAGAGAGTCTTATTCTCGAG AATAAGACTCTCTGATGATACTTTTTG | | | | | | | vector | |
| Dox-inducible-*shHdac2-2* | | CCGGACTGCATATTAGTCCTTCATTCTCGAG AATGAAGGACTAATATGCAGTTTTTTG | | | | | | | vector | |
| **Primers for RT-PCR** | | | | | | | | | | |
| Primer | Sequence (5' to 3') | | | | | | | | | |
| *Hdac1* | AGTCTGTTACTACTACGACG | | | | | | | Sense | | |
|  | TGAGCAGCAAATTGTGAGTCAT | | | | | | | Antisense | | |
| *Hdac2* | GGAGGAGGCTACACAATCCG | | | | | | | Sense | | |
|  | TCTGGAGTGTTCTGGTTTGTCA | | | | | | | Antisense | | |
| *Hdac3* | CACCAAGAGCCTTGATGCCTT | | | | | | | Sense | | |
|  | GCAGCTCCAGGATACCAATTACT | | | | | | | Antisense | | |
| *Hdac8* | GCAGCTGGCAACTCTGATT | | | | | | | Sense | | |
|  | GTCAAGTATGTCCAGCAACGAG | | | | | | | Antisense | | |
| *Oct-4* | TCTTTCCACCAGGCCCCCGGCTC | | | | | | | Sense | | |
|  | TGCGGGCGGACATGGGGA GATCC | | | | | | | Antisense | | |
| *Sox2* | GAGTGGAAACTTTTGTCCGAGA | | | | | | | Sense | | |
|  | GAAGCGTGTACTTATCCTTCTTCAT | | | | | | | Antisense | | |
| *endo-Oct4* | CCATGCATTCAAACTGAGGCACCA | | | | | | | Sense | | |
|  | AGCTATCTACTGTGTGTCCCAGTC | | | | | | | Antisense | | |
| *endo-Sox2* | GCACGGCCATTAACGGCACAC | | | | | | | Sense | | |
|  | CTCCATGCTGTTTCTTGCTGTCCTC | | | | | | | Antisense | | |
| *Nanog* | CAGGTGTTTGAGGGTAGCTC | | | | | | | Sense | | |
|  | CGGTTCATCATGGTACAGTC | | | | | | | Antisense | | |
| *Esrrb* | CATGAAATGCCTCAAAGTGGG | | | | | | | Sense | | |
|  | AAATCGGCAGGTTCAGGTAG | | | | | | | Antisense | | |
| *Rex1* | TCCAAGGAGCTGAACTCCT | | | | | | | Sense | | |
|  | CGTCTTGCTTTAGGGTCAGTT | | | | | | | Antisense | | |
| *Tet1* | GAGCCTGTTCCTCGATGTGG | | | | | | | Sense | | |
|  | CAAACCCACCTGAGGCTGTT | | | | | | | Antisense | | |
| *Klf4* | CTTCAGCTATCCGATCCGGG | | | | | | | Sense | | |
|  | GAGGGGCTCACGTCATTGAT | | | | | | | Antisense | | |
| *Epcam* | GCTGGCAACAAGTTGCTCTCTGAA | | | | | | | Sense | | |
|  | CGTTGCACTGCTTGGCTTTGAAGA | | | | | | | Antisense | | |
| *Cdh1* | AACAACTGCATGAAGGCGGGAATC | | | | | | | Sense | | |
|  | CCTGTGCAGCTGGCTCAAATCAAA | | | | | | | Antisense | | |
| *Ocln* | CCTCCAATGGCAAAGTGAATGGCA | | | | | | | Sense | | |
|  | TGTTTCATAGTGGTCAGGGTCCGT | | | | | | | Antisense | | |
| *Zeb1* | TGCTCACCTGCCCGTATTGTGATA | | | | | | | Sense | | |
|  | AGTGCACTTGAACTTGCGGTTTCC | | | | | | | Antisense | | |
| *Snail* | TTGTGTCTGCACGACCTGTGGAAA | | | | | | | Sense | | |
|  | TCTTCACATCCGAGT GGGTTTGGA | | | | | | | Antisense | | |
| *Sall4* | TGGTCCAGCCAATGACTCT TCCTT | | | | | | | Sense | | |
|  | TCGGATAAATGTTGGAGGGAGGCT | | | | | | | Antisense | | |
| *Cripto* | CAGTGCGTTTGAATTTGGACCCGT | | | | | | | Sense | | |
|  | AGTCCCTCCATTCAGACAGCAAGT | | | | | | | Antisense | | |
| *Tcl1* | TGGGAGAAGCACGTGTACTTGGAT | | | | | | | Sense | | |
|  | GTTGCCACATTAAAGGCAGCTCGT | | | | | | | Antisense | | |
| *Gapdh* | GTGTTCCTACCCCCAATGTGT | | | | | | | Sense | | |
|  | ATTGTCATACCAGGAAATGAGCTT | | | | | | | Antisense | | |
| *Actin* | GGCTGTATTCCCCTCCATCG | | | | | | | Sense | | |
|  | CCAGTTGGTAACAATGCCATGT | | | | | | | Antisense | | |
| *Fgf5* | TGCGTCCGCGATCCA | | | | | | | Sense | | |
|  | TCAGGGCCACGTACCACTCT | | | | | | | Antisense | | |
| *Dlx2* | CGGACAAGGAAGACCTTGAG | | | | | | | Sense | | |
|  | GGAGTAGATGGTGCGTGGTT | | | | | | | Antisense | | |
| *Mixl1* | ACTTTCCAGCTCTTTCAAGAGCC | | | | | | | Sense | | |
|  | ATTGTGTACTCCCCAACTTTCCC | | | | | | | Antisense | | |
| *Mesp1* | TGTACGCAGAAACAGCATCC | | | | | | | Sense | | |
|  | TTGTCCCCTCCACTCTTCAG | | | | | | | Antisense | | |
| *Lamina B1* | CCCCAATCTCTGTGAACCATG | | | | | | | Sense | | |
|  | GCAATTTGCACCGACACTGA | | | | | | | Antisense | | |
| *Sox17* | GGTCTGAAGTGCGGTTGG | | | | | | | Sense | | |
|  | TGTCTTCCCTGTCTTGGTTGA | | | | | | | Antisense | | |
| **Primers for genomic PCR genotyping** | | | | | | | | | | |
| Primer | Sequence (5' to 3') | | | | | | | | | |
| *Oct-4* | TCTTTCCACCAGGCCCCCGGCTC | | | | | Sense | | | | |
|  | GACGGCATCGCAGCTTGGATACAC | | | | | Antisense | | | | |
| *Sox2* | CCAATCCCATCCAAATTAACGC | | | | | Sense | | | | |
|  | GACGGCATCGCAGCTTGGATACAC | | | | | Antisense | | | | |
| *Klf4* | CTTCAGCTATCCGATCCGGG | | | | | Sense | | | | |
|  | GACGGCATCGCAGCTTGGATACAC | | | | | Antisense | | | | |
| *c-Myc* | TGACCTAACTCGAGGAGGAGCTGGAATC | | | | | Sense | | | | |
|  | GACGGCATCGCAGCTTGGATACAC | | | | | Antisense | | | | |
| *shHDAC2* | ATATACGATACAAGGCTGTTAGAGAG | | | | | Sense | | | | |
|  | CGAAGTCTCAGGAAGGCGGCAC | | | | | Antisense | | | | |
| **Primers for exogenous transgene silencing** | | | | | | | | | | |
| pMXs-TgUS | | | | GTGGTGGTACGGGAAATCAC | | | Sense | | | |
| pMXs-*Oct4*-TgDS | | | | TAGCCAGGTTCGAGAATCCA | | | Antisense | | | |
| pMXs-*Sox2*-TgDS | | | | GGTTCTCCTGGGCCATCTTA | | | Antisense | | | |
| pMXs-*Klf4*-TgDS | | | | GGGAAGTCGCTTCATGTGAG | | | Antisense | | | |
| pMXs-*c-Myc*-TgDS | | | | AGCAGCTCGAATTTCTTCCA | | | Antisense | | | |
| **Primers for ChIP analysis** | | | | | | | | | | |
| Primer | | | Sequence (5' to 3') | | | | | | | |
| *Nanog* promoter | | | TCCCTCCCTCCCAGTCTG | | | | | | | Sense |
|  |  |  | CCTCCTACCCTACCCACCC | | | | | | | Antisense |
| *Sall4* promoter | | | GGTCAGAGGAAGCTGCTATTG | | | | | | | Sense |
|  |  |  | GCCAGGGCTACACAGAGAA | | | | | | | Antisense |
| *Tcl1* promoter | | | GGAACTGTCTCCACTCCAACCA | | | | | | | Sense |
|  |  |  | TTGTGGCTCCATCGTTTGCT | | | | | | | Antisense |
| *Oct4* promoter | | | AATGAGTGATGTCGTGGGGTT | | | | | | | Sense |
|  |  |  | GAGGCTGCCCCTAAACTCTACGTA | | | | | | | Antisense |
| **Bisulfite Primers (5' to 3')** | | | | | | | | | | |
| *Oct4*-outside-forward | | | | | GAGGATTGGAGGTGTAATGGTTGTT | | | | | |
| *Oct4*-outside-reverse | | | | | CTACTAACCCATCACCCCCACCTA | | | | | |
| *Oct4*-inside-forward | | | | | CAAGCTTTGGGTTGAAATATTGGGTTTATTT | | | | | |
| *Oct4*-inside-reverse | | | | | CGGATCCCTAAAACCAAATATCCAACCATA | | | | | |
| *Nanog*- outside-forward | | | | | AAGTATGGATTAATTTATTAAGGTAGTT | | | | | |
| *Nanog-*outside-reverse | | | | | AAAAAACCCACACTCATATCAATATA | | | | | |
| *Nanog*-inside-forward | | | | | AAGTATGGATTAATTTATTAAGGTAGTT | | | | | |
| *Nanog*-inside-reverse | | | | | CAACCAAATCAACCTATCTAAAAA | | | | | |
| *Sall4*-outside-forward | | | | | ATTAAATTTAGGTAGGTAGATTTGAGAAATAAAT | | | | | |
| *Sall4*-outside-reverse | | | | | TTCAACTTTTCCCTCTAAAATCAA | | | | | |
| *Sall4-*inside-forward | | | | | TTAGGTAGATTTGAGAAATAAATTTTTATAT | | | | | |
| *Sall4-*inside-reverse | | | | | CTTTTCCCTCTAAAATCAAACCT | | | | | |
| *Tcl1*-outside-forward | | | | | ATTTGGTAGATTTGATTAGATGTTTA | | | | | |
| *Tcl1*-outside-reverse | | | | | TCCATCTACATATCTTCCTCCC | | | | | |
| *Tcl1*-inside-forward | | | | | AGTGAGAGGGTGAGAGGGAA | | | | | |
| *Tcl1-*inside-reverse | | | | | TCTCTCTATCACCTTCAATCTATCCT | | | | | |

**SUPPLEMENTAL TABLE S2**

| **Antibodies used in study** | | |
| --- | --- | --- |
| Antibodies | Catalogue and Lot Number | Application |
| HDAC1 | CST #2062 | Western |
| HDAC2 | Sc-7899 | Western |
| HDAC3 | BD 611124 | Western |
| HDAC8 | Ab39664 | Western |
| SSEA1 | Sc-101462 | Immunostaining |
| Nanog | Ab80892 | Immunostaining & western |
| GAPDH | Ab22556 | Western |
| Tublin | AP0013 | Western |
| Actin | Ab14128 | Western |
| HDAC2 | Ab7029 | ChIP & IP |
| TET1 | Sc-163443 | western |
| TET1 | GTX-124207 | IP |
| TET1 | Millipore #09-872 | ChIP |
| Ace-histone H3 | Millipore #06-599 | ChIP |
| RbAp46 | EPR5082 | IP |
| RbAp46 | EPR5083 | Western |
